# Esters of Mandelic Acid as a Basis for Chiral Eutectic Solvents: An Investigation of Physical Properties and Enantioselectivity

**DOI:** 10.1021/acs.jpcb.5c05034

**Published:** 2026-05-22

**Authors:** Olivia A. Gooch, Jenna N. Lane, Mandy Bechman, Valentine J. Klimkowski, Todd A. Hopkins

**Affiliations:** Department of Chemistry and Biochemistry, 4065 Butler University, 4600 Sunset Avenue, Indianapolis, Indiana 46208, United States

## Abstract

Deep eutectic solvents (DES) are mixtures of two or more components with a large depression in the freezing point due to strong intermolecular interactions such as hydrogen bonding. If one or more of the components are chiral, the DES is a chiral solvent that can be used in chirality-dependent applications, such as asymmetric catalysis or chiral separations. In this study, esters of mandelic acid were utilized as hydrogen bond donors (HBD) in the formation of 16 chiral DES with a variety of hydrogen bond acceptors. A common chiral HBD, (R/S) ethyl mandelate, is prepared in ionic DES with tetraalkylammonium (alkyl = ethyl, propyl, and butyl) chlorides, and tetrabutylammonium bromide and nonionic DES with thymol and 1,8-cineole. The density, viscosity, conductivity, and Kamlet–Taft parameters are measured and compared for each of the 16 chiral DES. The enantioselectivity of the ionic DES is measured by measuring the circularly polarized luminescence induced when a racemic mixture of lanthanide complexes is dissolved in the chiral DES. The thermodynamics show that the chiral DES with tetrabutylammonium bromide are most effective as enantioselective solvents. This correlates with the Kamlet–Taft measurements that show the bromide DES are the most effective hydrogen bond donors. Molecular dynamics (MD) simulations were performed for a subset of the DES to understand the molecular level interactions. MD results are analyzed to compare specific hydrogen bonding interactions in ionic and nonionic ethyl mandelate DES.

## Graphical abstract

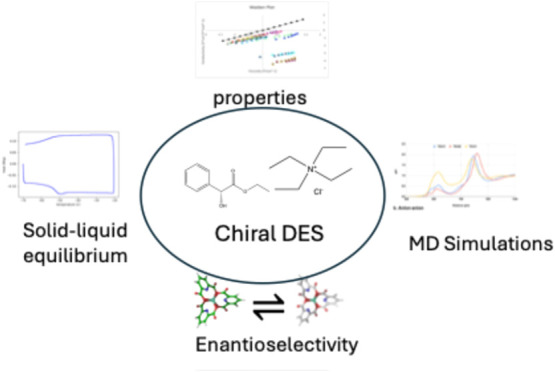

## Introduction

Deep eutectic solvents (DES) are mixtures of two or more components which have a melting point lower than an ideal mixture of the components.^1–3^ The melting point depression present in DES is the result of strong intermolecular interactions, such as Coulombic interactions, halogen bonding, or hydrogen bonding.^4–6^ Because hydrogen bonding is prevalent in many DES, the components of the mixture are typically labeled as hydrogen bond acceptors (HBAs) and hydrogen bond donors (HBDs). There is an established classification system, with DES categorized as type I–V based on the identity of the HBA and HBD.^2,7^ Type I, II, and IV are ionic DES that have ammonium (or phosphonium) salts as HBA mixed with metal chlorides or metal chloride hydrates as the HBDs.^2,8^ The most commonly used ionic DES are Type III, which have a quaternary ammonium salt as the HBA, most often choline chloride, mixed with an organic HBD.^1,6,9,10^ Type V DES are nonionic with organic HBA and HBD.^7,11–13^ Because they are typically prepared by simple mixing with 100% atom economy, DES are often considered as green solvents. The structural diversity of the HBA and HBDs offers a tunability of DES properties, such as conductivity, hydrophobicity, and viscosity, through the choice and ratio of HBA and HBD. Because of this tunability, DES are used in a range of applications, with a not exhaustive list that includes battery technology,^14–16^ pharmaceuticals,^17–21^ catalysis,^22–25^ and waste processing.^26–28^

When a DES is formed by mixing chiral molecules as the HBA and/or the HBD, the result is a chiral solvent with tunable properties that can be used in applications such as asymmetric synthesis^22–24,29,30^ and chiral separations.^31–34^ Chiral applications also include studies by our research group on the use of chiral DES as chiral light emitting materials.^35–38^ To further develop the use of chiral DES in chirality-dependent applications, it is important to characterize the relationship between HBA and HBD structure with physicochemical properties, including viscosity, conductivity and enantioselectivity. In a recent study, our research group screened ∼1700 mixtures of HBAs and chiral HBDs, identifying ca. 400 chiral DES or eutectic mixtures (ES).^39^ It was impossible to completely evaluate all 400 mixtures in that single study, but there are several chiral HBDs that formed liquids with many different HBAs leading to type III and V chiral DES. Two examples are the (R)- and (S)-enantiomers of ethyl mandelate, which formed DES or eutectic mixtures with all of the tetraalkylammonium salts (type III) and nonionic HBAs (type V) attempted in the database.^40^ In addition to the flexibility in forming DES, esters of mandelic acid can be sustainably produced chiral HBDs in DES. Mandelic acid is an ingredient in cosmetics and is isolated from almonds, and the esterification process of mandelic acid can be done catalytically with sustainably produced alcohols.^41,42^ This makes (R)/(S)-ethyl- and methyl mandelate the ideal chiral HBDs to study the impact of structure of the HBA on the properties of a chiral DES.

Table 1 shows the structures of the six HBAs and methyl and ethyl esters of mandelic acid that have been utilized as HBDs in this study. While the HBDs are structurally very similar, the HBAs include tetraalkylammonium salts with variable alkyl chain length (2–4 carbons) and halide anion to explore the impacts of cation and anion size on the properties of the corresponding type III DES. There are also two nonionic HBAs, thymol and 1,8-cineole, in this study to compare the properties of type III vs type V DES that have the same HBD. Because both thymol (thyme) and 1,8-cineole (eucalyptus) are isolated from biomass, these type V DES formed with the esters of mandelic acid could be considered a chiral solvent that meets the UN’s sustainable development goal 12 to “ensure sustainable consumption and production patterns”.^43^ Not all of the ionic and nonionic HBAs formed liquids with R- and S-MM, so only two mixtures with HBAs (TBACl and TBABr) are included in this study.

**Table 1:**
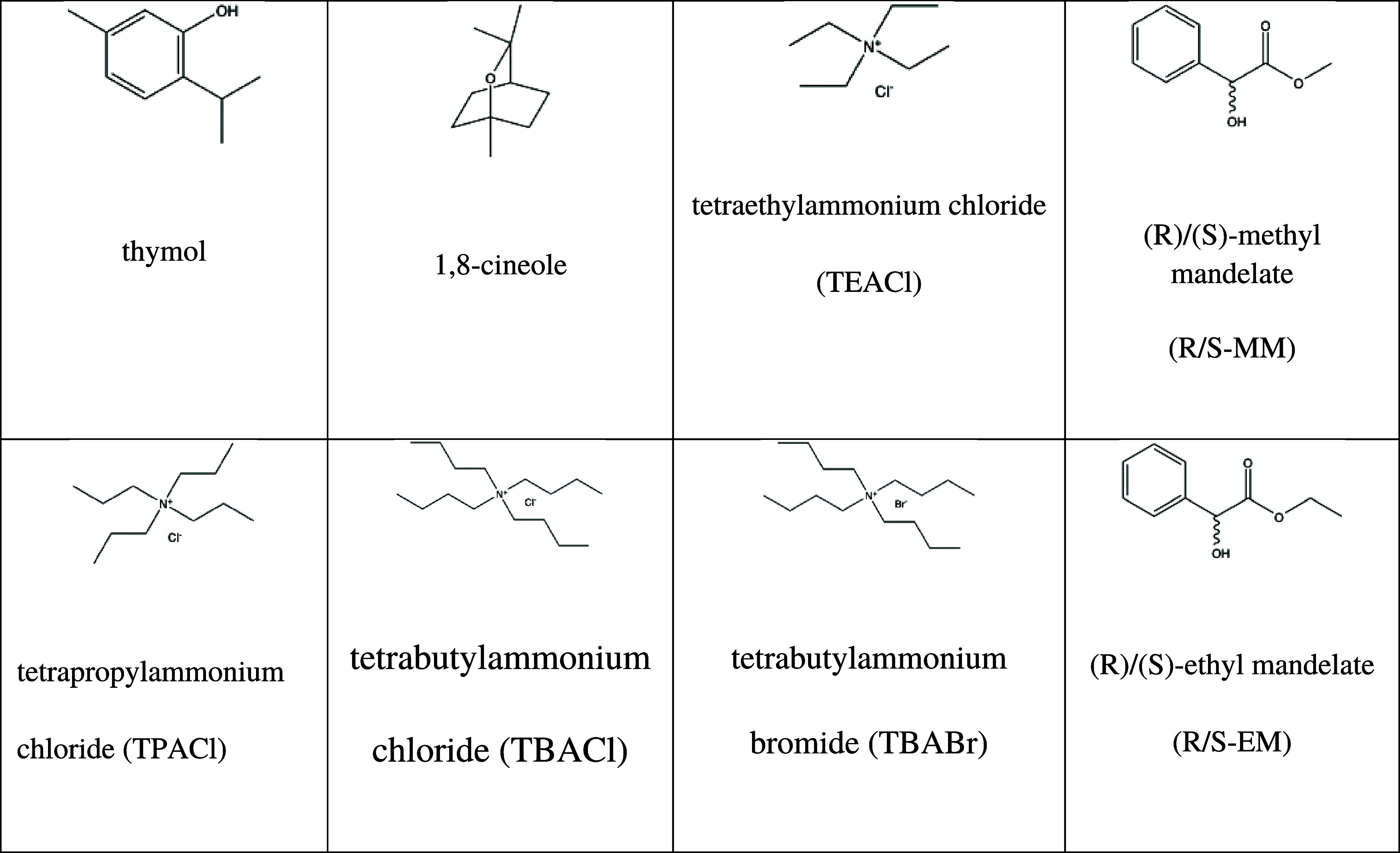
Structures of HBAs and HBDs

In this study, 16 mixtures at a 1:2 HBA:HBD mole ratio are prepared and the physical properties, including water content, density, viscosity, conductivity, thermal properties and the Kamlet–Taft parameters are measured. To understand and characterize the experimental properties of the DES on a molecular level, classical molecular dynamics (MD) simulations were run for nine of the mixtures. Eight of the simulated DES had ethyl mandelate as the HBD with three ionic HBAs, including TBACl, TBABr, and TEACl, and nonionic HBA, thymol. These simulations probe the impact of cation size, anion identity, and ionic vs nonionic HBA on the liquid properties of the DES. The other DES simulated was 1:2 TBACl: (R)-MM to compare the impact of changing the HBD, MM vs EM. Molecular dynamics simulations are effective for characterizing how varying the components affect the structure and hydrogen bonding network of DES, including studies that evaluate the impact of cation size,^44–47^ anion,^47,48^ and HBD.^49–51^ Analysis of the simulations of the nine DES provides information about the structure and hydrogen bonding network for EM DES with different HBAs. Results from the MD simulations will be compared to experimental properties. The ability of the chiral DES to exhibit enantioselectivity is measured through circularly polarized luminescence (CPL) spectra of a luminescent lanthanide complex, Eu­(dpa)_3_^3–^ (where dpa = 2,6-pyridine dicarboxylate anion) added to the DES. CPL is the differential emission of left vs right circularly polarized light. In the absence of a chiral perturbation, Eu­(dpa)_3_^3–^ exists as an interconverting racemic mixture of Λ- vs Δ-enantiomers, which would give a CPL signal of zero. When added to a chiral solvent, such as these chiral DES, the Eu­(dpa)_3_^3–^ population becomes nonracemic resulting in a nonzero CPL signal.^35,52^ The sign and magnitude of the CPL signal is a measure of the handedness and degree of enantioselectivity demonstrated by the solvent. Temperature dependent CPL spectra of Eu­(dpa)_3_^3–^ in the ten ionic chiral DES provide a measure of the thermodynamics of enantioselectivity. Since Eu­(dpa)_3_^3–^ is insoluble, it is impossible to measure the enantioselectivity of the two nonionic type V chiral DES.

## Experimental Section

### DES Preparation

The chemicals used in this study, (R)- and (S)-ethyl mandelate, (R)- and (S)-methyl mandelate, tetrabutylammonium chloride, tetrapropylammonium chloride, tetraethylammonium chloride, tetrabutylammonium bromide, thymol, 1,8-cineole, Nile red, 4-nitroaniline, and *N*,*N*-diethyl-4-nitroaniline, were purchased from Combi-blocks and/or VWR and used without further purification. The tetraalkylammonium salts were stored in a vacuum desiccator before use in mixture preparation. Mixtures were prepared by adding the correct molar ratios of hydrogen bond acceptor and hydrogen bond donor to a sample vial and stirring under heat at temperatures <50 °C until a homogeneous liquid was formed (typically less than 1 h). Several of the mixtures were dried in a vacuum desiccator for 1 week before use.Water content of the mixtures was measured by volumetric Karl Fischer titration (Metrohm 870 KF Titrino Plus).

### Water Content

Upon initial preparation under ambient conditions, the mixtures with TBABr, thymol, or 1,8-cineole contained <1 wt % of water. However, several of the mixtures with tetraalkylammonium chloride HBDs were 1–3 wt % water under ambient conditions. Most of the samples were <1 wt % water when dried in a vacuum desiccator for <4 h, but TEACl, TPACl and two TBACl DES required drying in a vacuum desiccator for 5–7 days to get to <1 wt % water. The mole fractions including water for the 1:2 HBA:HBD mixtures are shown in Table S1. Where applicable, the table includes both the partially dried and more completely dried (shown in parentheses) mole fractions for the six Cl^–^ DES. To more closely represent conditions in which the DES would be used, the Kamlet–Taft, conductivity and enantioselectivity measurements were measured for the DES samples prepared with <4 h in a vacuum desiccator. Therefore, the six Cl^–^ DES have water mole fractions between 0.16 and 0.25, which is relatively high but within a range to be considered “water in DES”.^53^ To enable a better comparison across DES samples, the density and viscosity measurements are reported on the DES samples dried for 5–7 days. For simplicity of language, the mixtures will be labeled as molar ratios of HBA:HBD without quantifying the water content.

### Physical Measurements

The melting points of mixtures were measured with a differential scanning calorimeter (DSC) (TA Instruments DSC 25). DSC samples were prepared by adding 3–10 mg of the mixture to an aluminum pan and lid. The DSC was operated with a typical heating and cooling rate between 2 and 5 °C/min under a constant flow of nitrogen gas. Each sample was run through at least two cooling–heating cycles. The water content of the larger mass samples was measured immediately before measuring the density, viscosity, and conductivity. Densities were obtained by determining the mass of DES in a 1.105 mL glass pycnometer. The viscosity was measured with a Brookfield DV2T viscometer and conductivity was measured using a conductivity meter (Thermo Scientific Orion Star A212). Conductivities and viscosities were measured over a 283–323 K temperature range using a circulating water bath to control temperature. Kamlet–Taft parameters were determined by dissolving small quantities of the solvatochromatic dyes, Nile red, 4-nitroaniline, and *N*,*N*-diethyl-4-nitroaniline, in each of the DES and measuring the UV–vis spectra (Cary 60).

### Spectroscopy Measurements

All of the DES samples evaluated for CPL were molar ratio, 1:2 HBA:HBD. CPL samples were prepared by dissolving small quantities of [TBA]_3_Eu­(dpa)_3_ in 1–2 g of DES to give concentrations of (1–4) × 10^–6^ molal. The CPL of Eu­(dpa)_3_^3–^ samples dissolved in the DES was measured using a custom-built spectrometer described previously.^37,38^ A jacketed cuvette holder with water circulating was used to control the temperature for temperature dependent CPL measurements over 10–45 °C range. Luminescent lifetimes of the Eu­(dpa)_3_^3–^/DES samples were measured on a custom-built instrument described previously.^37,38^

### Simulation Methods

Of the 16 potential DES listed in Table S1, we performed MD simulations for a subset of nine. Each system has a 1:2 mol ratio of HBA to HBD. Specifically, eight utilized the HBD EM (R and S-isomers), and one the HBD MM (R-isomer only). For those six systems containing the HBD EM and a tetraalkylammonium HBA (TBACl:(R/S)­EM; TEACl:(R/S)­EM and TBABr:(R/S)­EM), two separate systems were constructed containing a distinct 3d isomer structure of the Eu­(dpa)_3_^3–^ complex, either Λ- or Δ-isomer, along with three sodium ions. No analysis of the behavior of the Eu­(dpa)_3_^3–^ complexes dissolved in the DES is provided in this study, and the presence of one complex should have little effect on the simulations of the DES. Finally, for all constructed systems, the equivalent number of water molecules corresponding to the experimental mole fraction of water (Table S1) was included. Each DES system contains explicitly 100 HBA and 200 HBD molecules, along with the appropriate number of other components as detailed in Table S6. In total 15 3-dimensional (3D) systems were constructed, and 17 equiv MD simulations were performed. The latter includes repeating the MD for the thymol systems with a different initial random number seed. The starting configuration for each was built using Packmol (version 20.3.3) as a cube with initial side length determined for an assumed starting density of 1.0 g/mL (Table S6).^54^ The 2D structure of each molecular component is shown in Table 1. A representative 3D structure of each component, along with associated abbreviations and atom numbering, is illustrated in Figure S16.

The NAMD2 software (version 2.14 for Linux-x86_64-multicore) was used to perform the MD simulations.^55,56^ The CHARMM style force field^57^ was used with parametrization for the CHARMM General Force Field (CGenFF, version 4.6).^58,59^ For the TEA and TBA cations, Thymol and the EM and MM HBD components, the CGenFF Web site facilities (version 2.2.0) were used to build a CHARMM format topology file, and assign atom types, parameters and charges.^60,61^ The combined topology and parameter data structures for each are listed in the Supporting Information. Previous validation of the CGenFF for determining condensed phase pure solvent properties appear in the literature.^58,62^ The CHARMM chloride anion and sodium ion parameters were used, while the corresponding bromide anion parameters were taken from Joung and Cheatham.^63^

For MD simulations, periodic boundary conditions (PBC) were used and initially defined with respect to the specific cubic side length of each DES system (Table S6). Standard CHARMM energy evaluation settings were used. To truncate and smooth all nonbonded interactions, a switching function (force-based switching function for LJ interactions) was used within a 10–12 Å switching range. However, the nonbond pair list was maintained out to 16 Å and reevaluated every 10 fs. Additionally, the Particle Mesh Ewald method was used to treat long-range electrostatic interactions.^64^

The initial configuration of each system was first subjected to 20,000 steps of conjugate gradient minimization. All subsequent MD was performed using a 1 fs time step while fixing the length of all bonds containing a H atom using the SHAKE algorithm.^65^ The first stage of MD consisted of 1 ns early equilibration at room temperature (293.15 K) performed using the NVT ensemble by reassigning the velocities to 293.15 K every 100 fs. This was followed by a next stage of 8 ns NPT equilibration where the PBC cubic side length was allowed to vary to maintain a pressure of 1.01325 bar (1 atm). The system volume from the last 3 ns of this stage was plotted to evaluate stability of the simulation. In each case, the average volume from the last 0.5 ns of this stage was used to derive a new PBC cubic side length representing the average density for each system at room temperature. Next, using the revised PBC settings, 2 ns further late NVT equilibration was performed where the temperature was checked every 500 fs to ensure no drift occurred. During equilibration the pressure was controlled using a semi-isotropic Nosé–Hoover Langevin-piston method with a piston period of 50 fs and a piston decay of 25 fs.^66,67^ Throughout, the 293.15 K temperature was maintained using Langevin temperature coupling with a friction coefficient of 1 ps^–1^. Finally, 100 ns of NVT production dynamics were performed.

For a subset of the systems (TBACl:(R/S)-EM; TEACl:(R/S)-EM; TBABr:(R/S)-EM; Thymol:(R/S)-EM), we repeated the MD simulations using exactly the same procedure outlined, but with the following changes. During the equilibration stages the 293.15 K temperature was maintained using the stochastic velocity rescaling method of Bussi with a temperature coupling period of 1 ps applied at an increasingly less frequent interval (20 to 1000 timesteps).^68^ Additionally, these simulations did not include the three-dimensional isomer structure of the Eu­(dpa)_3_^3–^ complex and the three sodium counterions. The 100 ns production dynamics results of these additional simulations were used solely to derive the hydrogen bond lifetime properties as applied previously for similar systems.^47,69,70^

During every MD stage all energy components were written to the log file every 250 fs. Subsequently, various Linux shell scripts were used to extract out and postprocess the energy components to both monitor the behavior of the system (temperature, and potential/kinetic energies) and to derive additional properties (density). Likewise, during the MD production stage the position coordinates were written to the binary trajectory file every 500 fs. Afterward these trajectories were converted to XYZ format ASCII files using the CatDCD utility (version 4.0), each containing 200,000 sequential coordinate sets. It is these trajectory files that were used for analyzing the structural solution properties using the TRAVIS software (version Jul 29 2022).^71,72^ Miscellaneous additional analysis and visualization was done with VMD software (version 1.9.4a57-arm64-Rev12).^73^

## Results and Discussion

### Thermal Properties

Differential scanning calorimetry was used to measure the freezing/melting temperatures of the mixtures of HBA with methyl- and ethyl-mandelate. For almost all mixtures at 1:2 HBA:EM/MM, the DSC only showed a low temperature glass transition (Figure S7). Attempts to induce thermodynamic crystalline freezing/melting in the mixtures with TEACl, TPACl, and TBACl anywhere near a eutectic point were unsuccessful. Many of these mixtures have higher mole fractions of water that complicate the interpretation of the observed melting transitions. Only mixtures of TBABr, thymol, and 1,8-cineole with (S)-EM were completely characterized in terms of the solid–liquid equilibrium (SLE) curve. Mixtures of 1,8-cineole with (S)-EM did not show a v-shaped SLE phase diagram (Figures S5 and S6), which may indicate cocrystallization or polymorphism.^74^ The mixtures of TBABr and thymol with (S)-EM showed a melting transition or an experimentally observed solidus transition that helped to identify the eutectic point.

Figure 1 shows the SLE curves for TBABr mixed with (S)-EM (Figure 1a) and thymol mixed with (S)-EM (Figure 1b). Each of these mixtures has a relatively low water content, measuring less than 3 mol % of water in a 1:2 ratio sample (Table S1), but water content was not measured for each of the DSC samples. Thus, the mole fraction of (S)-ethyl-mandelate on the *x*-axis in Figure 1 is an approximation based only on the HBA:HBD binary mole fraction. In each of the plots, there are gaps in the SLE curves due to the inability to observe thermodynamic crystallization melting/freezing over some of the molar ratios. For example, Figure 1b shows only two data points at x_thymol_ < 0.5, because the DSC plots only showed low temperature, ∼215 K, glass transitions. In the TBABr:(S)-EM mixture, this prevents the observation of the melting point at the eutectic point. A low temperature melting transition appears at 266 K in the *x*_EM_ = 0.875 and 0.800 DSC thermograms (Figure S3), which can be attributed to the solidus melting transition. Since the temperature of the solidus line should match the melting temperature at the eutectic point,^2^ the eutectic point can be estimated in the range between *x*_EM_ = 0.6–0.7. Figure 1b shows the measurable SLE curve for thymol and (S)-EM. There was no thermodynamic melting observed between *x*_EM_ = 0.2–0.5, where only low temperature glass transitions are observed (Figure S4). Based on Figure 1b, the eutectic point of thymol:(S)-EM appears to be at *x*_EM_ = 0.66.

**Figure 1:**
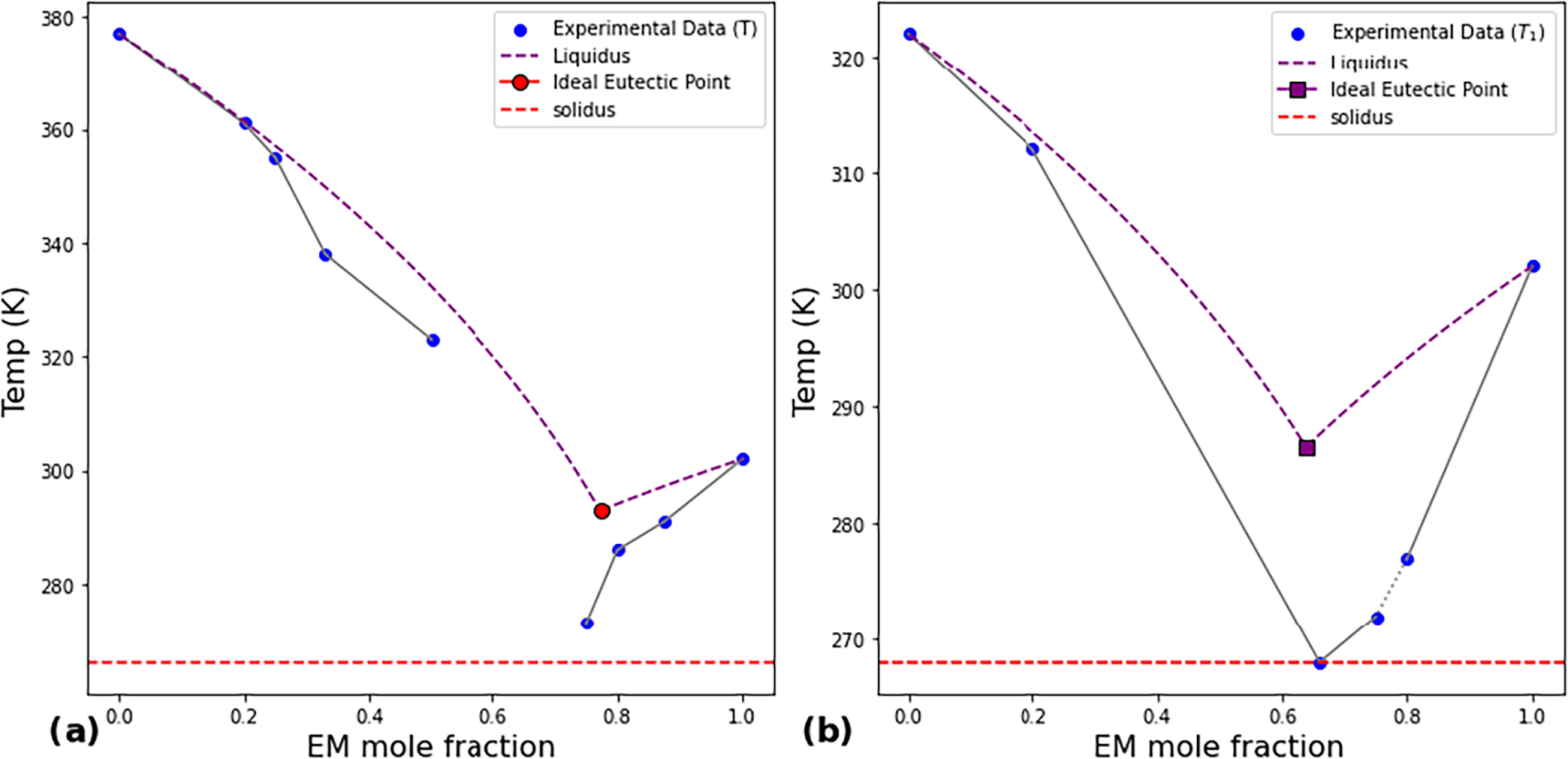
Solid liquid equilibrium curves for mixtures of (a) TBABr and (S)-EM, and (b) thymol and (S)-EM. The red dotted line is the experimental solidus line at (a) 266 K and (b) 268 K. The gray line represents the liquidus line. The purple and green dotted lines represent the liquidus lines for ideal mixtures. In (a) TBABr:(S)-EM, no crystallization was observed between *x*_EM_ = 0.5–0.80, where the thermograms only showed glass transitions ∼220 K (see Figure S3). Only glass transitions were observed for thymol:(S)-EM between *x*_EM_ = 0.2–0.66 (see Figure S4).

To evaluate the thermodynamics of mixing, the ratios, 1:2 TBABr:(S)-EM and 1:2 thymol:(S)-EM, can be used in eq 1 to determine the activity coefficients of the mixtures.

1 $$\ln\left(\gamma_{A}x_{A}\right)=\frac{{\Delta H}_{fus}}{R}\left(\frac{1}{T_{fus}}-\frac{1}{T}\right)$$

where γ_A_ and *x*_A_ are the activity coefficient and mole fraction of component A, *R* is the gas constant, *T*_fus_ and Δ*H*_fus_ are the melting point and enthalpy of fusion of the component A, and *T* is the melting point of the mixture. This equation assumes that the contribution from the change in heat capacity upon melting (Δ_fus_ *C*_p_) is negligible.^75^ The Δ*H*_fus_ and *T*_fus_ for TBABr and thymol were previously reported in the literature,^76,77^ but to the best of our knowledge ethyl mandelate was not. The *T*_fus_ for (S)-EM was determined by DSC, and Δ*H*_fus_ by integrating the DSC peak (Figure S2). The thermodynamic values used in eq 1 are shown in Supporting Information, Table S2. According to Figure 1b, the 1:2 ratio of thymol:(S)-EM is the eutectic point, and the activity coefficients determined at this ratio using eq 1 were γ_EM_ = 0.528 and γ_thymol_ = 0.578 showing the nonideality in this mixture. Whether a mixture is a DES can be determined using a dimensionless molar excess Gibbs free energy proposed by Panzer,^78^ which is shown in eq 2

2 $$\frac{G^{E}}{\mathrm{RT}}=\sum x_{i}{\ln\gamma }_{i}$$

where *x_i_* is the mole fraction and γ*_i_* is the activity coefficient of the *i*th component, *G* ^E^ is the molar excess Gibbs free energy. For 1:2 thymol:(S)-EM, the *g* ^E^/*RT* = −0.60 (eq 2) which is less than the −0.33 threshold used to distinguish a “true” DES. Assuming that the solidus temperature is the melting point and an approximation of 1:2 ratio of TBABr:(S)-EM (Figure 1a) as the eutectic point in eq 1 gives γ_EM_ = 0.492 and γ_TBABr_ = 0.353, which results in *g* ^E^/*RT* = −0.81 (eq 2). Therefore, by the quantitative thermodynamic data, both of these mixtures are DES.

The lack of melting transitions at similar molar ratios makes it difficult to determine the eutectic point or to identify the other mixtures as DES. Figure S7 shows DSC thermograms for all of the other mixtures including mixtures with (R)-EM as HBD, which shows that the melting transitions are independent of the handedness of the HBD. A 1:2 HBA:HBD ratio is the experimentally determined eutectic point for TBABr:EM and thymol:EM, and therefore these are the only mixtures that can be identified as DES. The remaining mixtures exhibit similar low-temperature glass transitions at a 1:2 molar ratio (Figure S7), but lack the evidence that they are DES or ideal mixtures (eutectic solvents). For simplicity of language, all of the mixtures in this study will be referred to as DES.

### Density, Viscosity, and Conductivity

Figure 2 shows the densities of 12 DES of 1:2 HBA: (R)- vs (S)-EM and (R)-MM vs (S)-MM at 293 K. A full table of the measured densities and viscosities at 293 K is provided in Supporting Information (Table S3). Given that the HBAs are achiral, it is not surprising that there is not a significant enantiomeric effect in the density between the (R)- vs (S)-EM DES. Comparison across the tetraalkylammonium chloride DES series shows that the density decreases as the alkyl chain increases, indicating closer packing for the smaller cations. Comparison of TBABr vs TBACl with both EM and MM HBDs shows that the density increases for Br^–^ vs Cl^–^. Comparison of EM vs MM shows no difference with TBABr as the HBA, but the 1:2 TBACl:MM DES are ∼5% more dense than 1:2 TBACl:EM DES. While MM has a smaller molar volume than EM, the density results for the DES suggest that the HBD size difference is not the sole determining factor.

**Figure 2:**
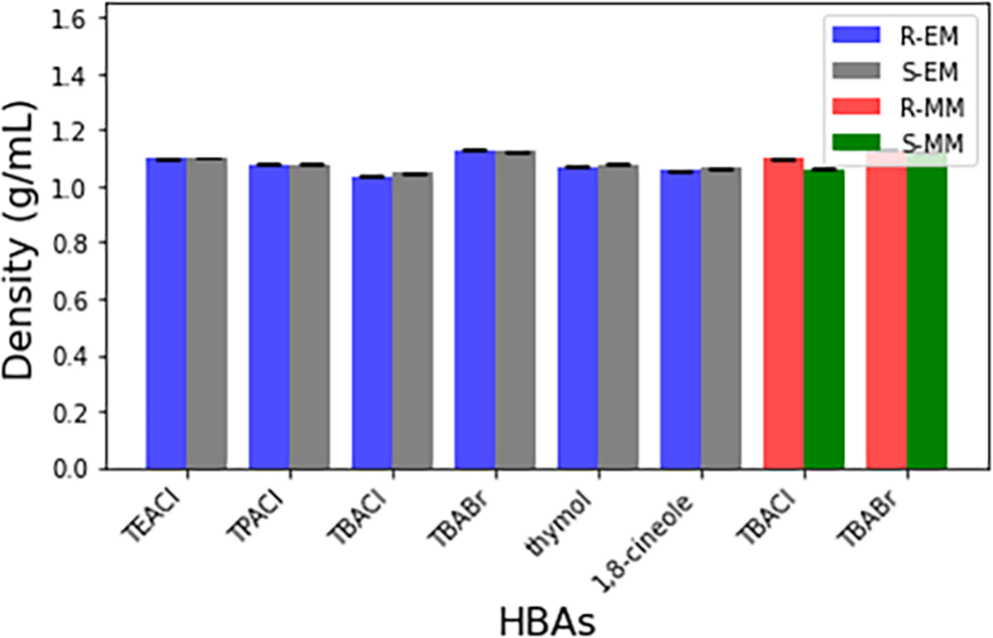
Densities of 1:2 HBA:(R)- (blue bars) vs (S)-EM (gray bars) and 1:2 HBA:(R)-MM (red bars) vs (S)-MM (green bars) at 293 K. Error bars represent the uncertainty of ±0.004 g/mL.

The viscosity was measured for all 16 DES over a 288–318 K temperature range, and Table S3 lists the viscosity at 293 K. The nonionic DES, thymol and 1,8-cineole with (R)- and (S)-EM have the smallest viscosities of the measured DES by an order of magnitude (20–30 mPa-s at 293 K). The viscosities of the ionic DES are moderately high, 100–1000 mPa-s range with the largest viscosity, 950 mPa-s for 1:2 TBABr:MM.

Figure 3 shows the temperature dependent viscosity for the dried samples of 1:2 TEACl, TPACl, TBACl, and TBABr:(R)-/(S)-EM over a 288–318 K temperature range. The remaining temperature dependent viscosities are shown in Figures 4 and S9. Each of the plots show an Arrhenius-like exponential decay of viscosity with temperature. Like the density, there is very little difference in viscosity based on the stereoisomer of EM with the exception of the two lowest temperature, 288 and 293 K, measurements where TBABr:(R)-EM is greater than TBABr:(S)-EM (Figure 3b). The water mole fractions (Table S1) in both DES are small with (R)-EM having higher water content. Since water would be expected to reduce the viscosity, the sample water content does not explain the difference. Previous studies have shown that the viscosity increases with increasing cation chain length for tetraalkylammonium DES.^79^ Figure 3a,3b shows a viscosity trend of TEACl < TBACl < TPACl, although there is not a large difference in the viscosities of TPACl vs TBACl. Figure 3b also shows that the 1:2 TBACl: (R)/(S)-EM have slightly higher viscosities than 1:2 TBABr:(R)/(S)-EM. Figure 4a shows the temperature dependent viscosities for 1:2 TBACl: vs TBABr:(R)/(S)-MM, where the TBABr:MM viscosity is slightly higher than TBACl:MM at the lowest temperatures. While there is no clear trend, any impact of Cl^–^ vs Br^–^ on the viscosity of these DES is small.

**Figure 3:**
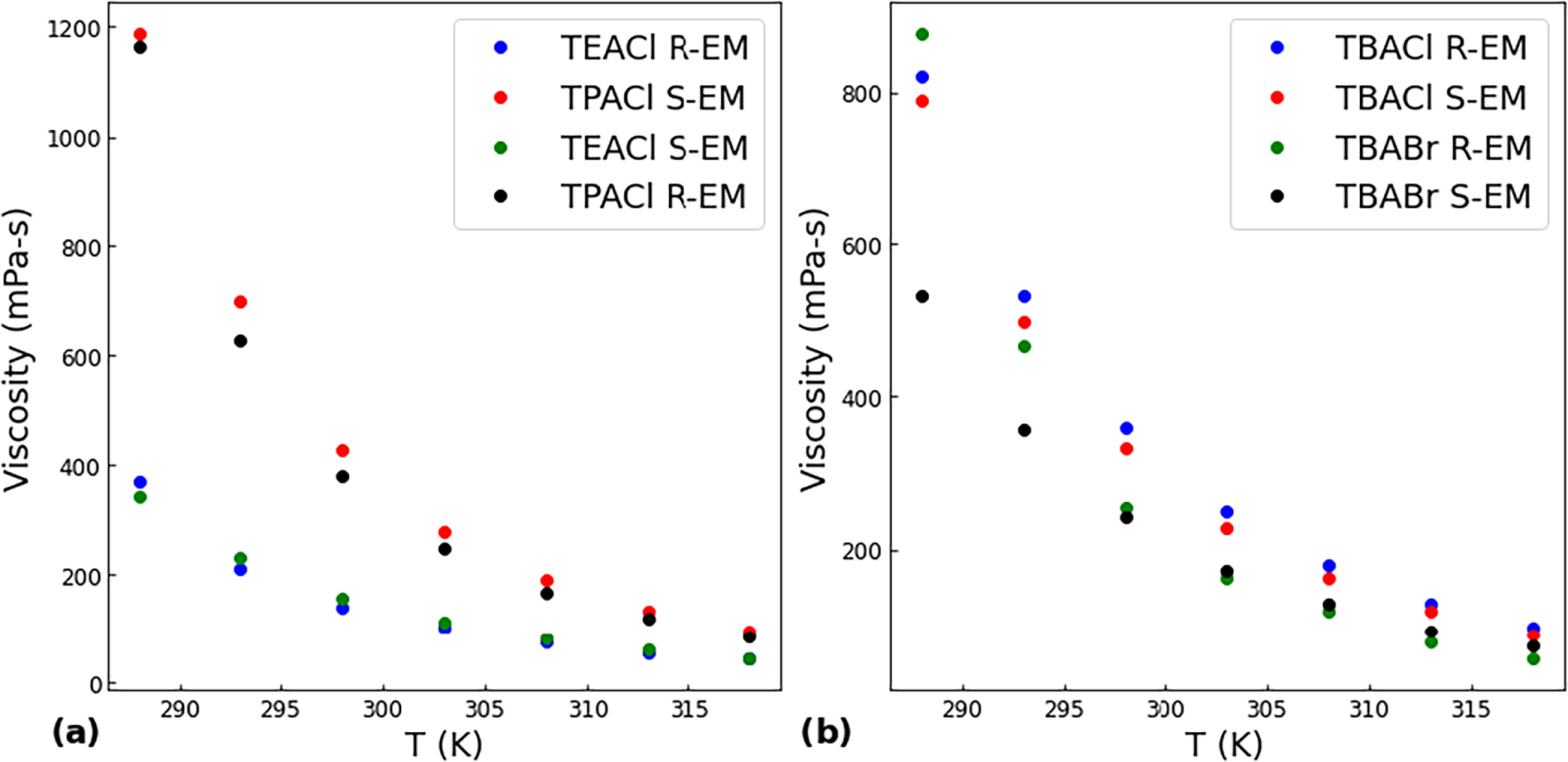
Viscosity vs temperature for (a) 1:2 TEACl:(R)-EM (blue), 1:2 TEACl:(S)-EM (green), 1:2 TPACl:(R)-EM (black), 1:2 TPACl:(S)-EM (red), and (b) 1:2 TBACl:(R)-EM (blue), 1:2 TBACl:(S)-EM (red), 1:2 TBABr:(R)-EM (green), and 1:2 TBABr:(S)-EM (black).

**Figure 4:**
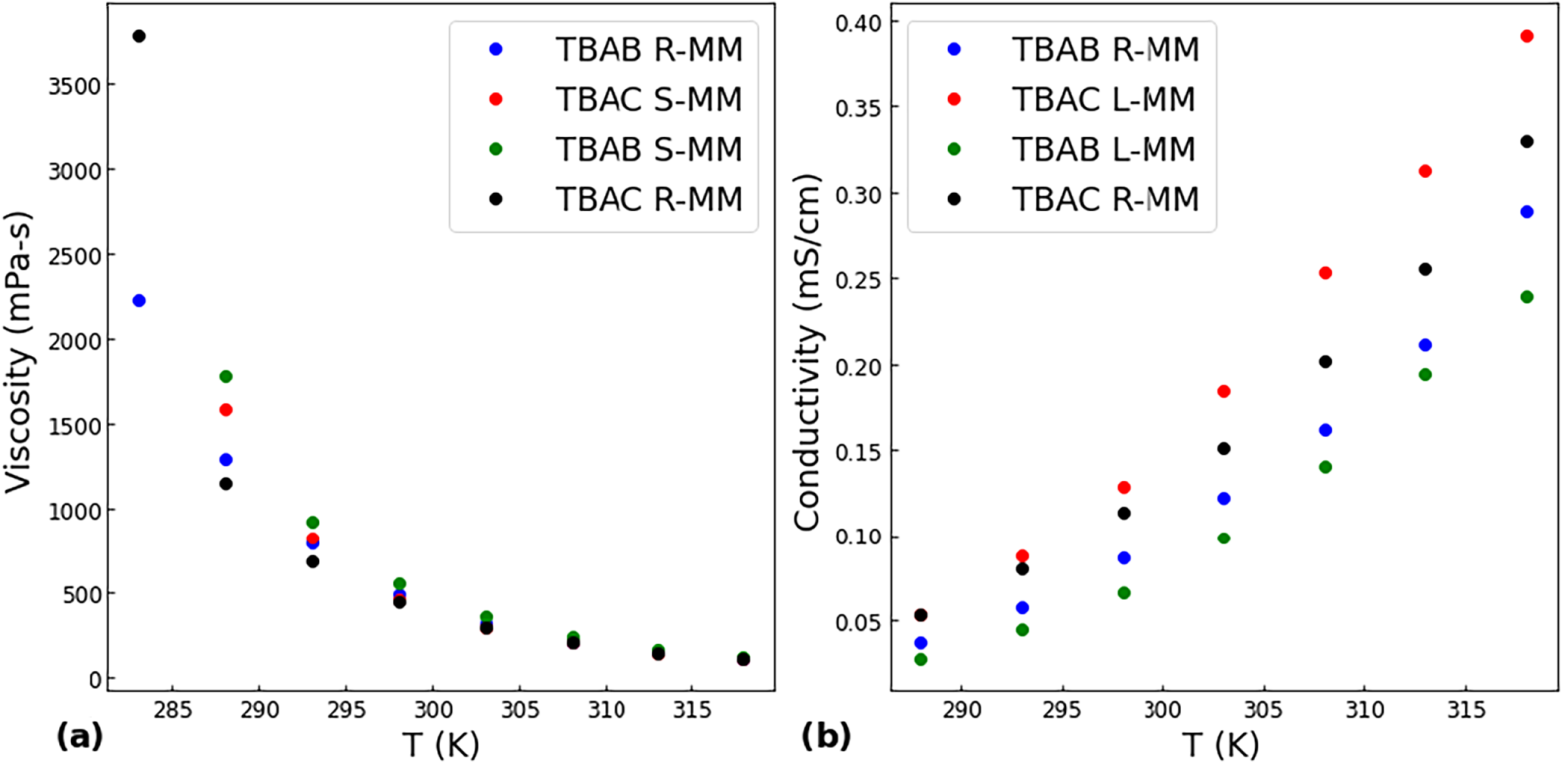
(a) Viscosity vs temperature and (b) conductivity vs temperature for 1:2 TBABr:(R)-MM (blue), 1:2 TBABr:(S)-MM (green), 1:2 TBACl:(R)-MM (black), and 1:2 TBACl:(S)-MM (red).

Figure 4b shows the temperature dependent conductivities for 1:2 TBACl: vs TBABr:(R)/(S)-MM over 283–318 K. The conductivities for the remaining ionic DES are shown in Figure S8. For the tetraalkylammonium chloride EM DES, the conductivities are inversely proportional to the viscosity. However, the conductivities of TBACl:EM and MM are higher than TBABr:EM and MM, respectively. Since there was very little difference in the TBACl vs TBABr viscosities, this suggests that the Cl^–^ DES are more conductive than the Br^–^ DES. The conductivities of the nonionic DES, thymol: EM and 1,8 cineole:EM, were too low to accurately measure.

### Kamlet–Taft Solvent Parameters

The solvation behavior of the DES was measured using three solvatochromatic dyes to determine the Kamlet–Taft (K–T) parameters.^80–82^ Table 2 shows the K–T parameters measured for all eight of the DES with (R)-EM or (R)-MM (shown without the chirality label). The (S)-EM and -MM DES are not included in this table because there was no difference within uncertainty in the K–T parameters for R vs S-EM. A complete table with K–T parameters for 14 of the DES is in the Supporting Information (Table S4). For most DES, except with thymol, the only hydrogen bond donation (acidity) comes from the hydroxyl group in ethyl- or methyl-mandelate. Comparison of TBACl and TBABr: MM vs EM in Table 2 shows that methyl-mandelate DES are better hydrogen bond donors, larger α, than the ethyl-mandelate DES, and the hydrogen bond basicity (β) and polarizability (π*) are both larger for EM vs MM DES. This also shows that α and β are higher for TBABr vs TBACl. This indicates that the “weaker” hydrogen bonding in the Br^–^ DES results in stronger hydrogen bond interactions with the solute.

**Table 2:** Kamlet–Taft Parameters for the EM and MM DES

DES^a^	α^b^	β^b^	π*^b^
TBABr:EM	0.74	1.23	1.29
TBACl:EM	0.54	1.16	1.11
TPACl:EM	0.54	1.12	1.12
TEACl:EM	0.44	1.04	1.34
TBACl:MM	0.64	0.73	0.96
TBABr:MM	0.84	0.84	0.89
Thymol:EM	0.72	1.16	1.06
1,8-cineole:EM	0.42	1.20	1.02

a All DES at a 1:2 HBA:HBD mole ratio

b uncertainty in α and β = ±0.12, and π* = ±0.06.

The data in Table 2 also shows that the size of the alkyl chain has very little impact (slight increase for larger alkyl group) on α, whereas π* decreases and β increases with increasing alkyl chain length. Zhang et al. observed these same trends with respect to alkyl chain length for DES with ethylene glycol as HBD.^83^ Comparison of the two nonionic DES (Table 2) shows similarity in β and π* with the ionic EM DES, which indicates that the EM plays a significant role in determining these parameters, The thymol:EM has a larger hydrogen bond acidity (α) than 1,8-cineole:EM because thymol has an additional hydroxyl group for hydrogen bond donation. The α for thymol:EM is consistent with the α measured by Schaeffer et al. for several thymol DES, but the β and π* shown in Table 2 are larger than was typical for thymol DES.^84^

### Molecular Dynamics Results

Molecular dynamics simulations were run on a subset of the chiral EM-based DES (Table S6) and analyzed to try to understand how changing the cation size, anion, and ionic vs nonionic mixtures affect the molecular-level interactions (where the MM-based DES lacked the HBA diversity for this analysis). Simulations were run for eight (R)- vs (S)-EM DES with TEACl, TBACl, TBABr, and thymol as HBAs. In order to validate the simulations, the experimental and simulated densities were compared for the eight DES (shown in Table S7). The densities show that the simulated densities are lower than experiment, but the differences are small (2–7%). The analysis of the simulations for the DES showed no chiral dependence, the radial distribution function analysis includes an average of (R)- and (S)-EM simulation results. Since the EM-EM atom-specific RDFs (Figure S19) show only one hydrogen bond radius, this indicates that the R–R and S–S radii are equivalent, and the lack of a second short-distance radius (R–S) indicates that enantiomer interconversion is not occurring over the course of the simulations. The experimental physical properties, such as density and K–T parameters, show no enantiomer-dependence, averaging the enantiomeric simulation results provides a relevant comparison to experiment.

Figure 5 shows the atom specific RDFs involved in the hydrogen bonding present in four of the DES, including 1:2 TBABr/TBACl/TEACl/thymol:EM, derived from the analysis of MD. Figure 5a shows the RDFs for the hydrogen bonding interaction between the hydroxyl hydrogen (H9) on the ethyl mandelate with the anion (ionic HBAs) or the hydroxyl oxygen of thymol. Figure 5b,c show the atom-specific RDFs of the hydroxyl oxygen (O2) and carbonyl oxygen of the ester (O3) of EM interacting with the average of the alkyl hydrogens that are closest to the nitrogen in TBA^+^ and TEA^+^, collectively labeled H_inner_ (i.e., over the time evolution of the simulation, all of these alkyl H’s are involved in hydrogen bonding). Even though these hydrogens are bonded to carbon, this short-range interaction (∼200 pm) is consistent with a hydrogen bonding interaction, and a previous study has demonstrated this in other tetraalkylammonium-based DES.^47^ Not only does the anion of the HBA act as a hydrogen bond acceptor (Figure 5a) but the cation of the HBA acts as a hydrogen bond donor in the formation of DES with tetraalkylammonium halides and EM. In the nonionic DES, Figure 5b,c shows the atom-specific RDFs of the H5 (thymol) with the O2 and O3 of EM.

**Figure 5:**
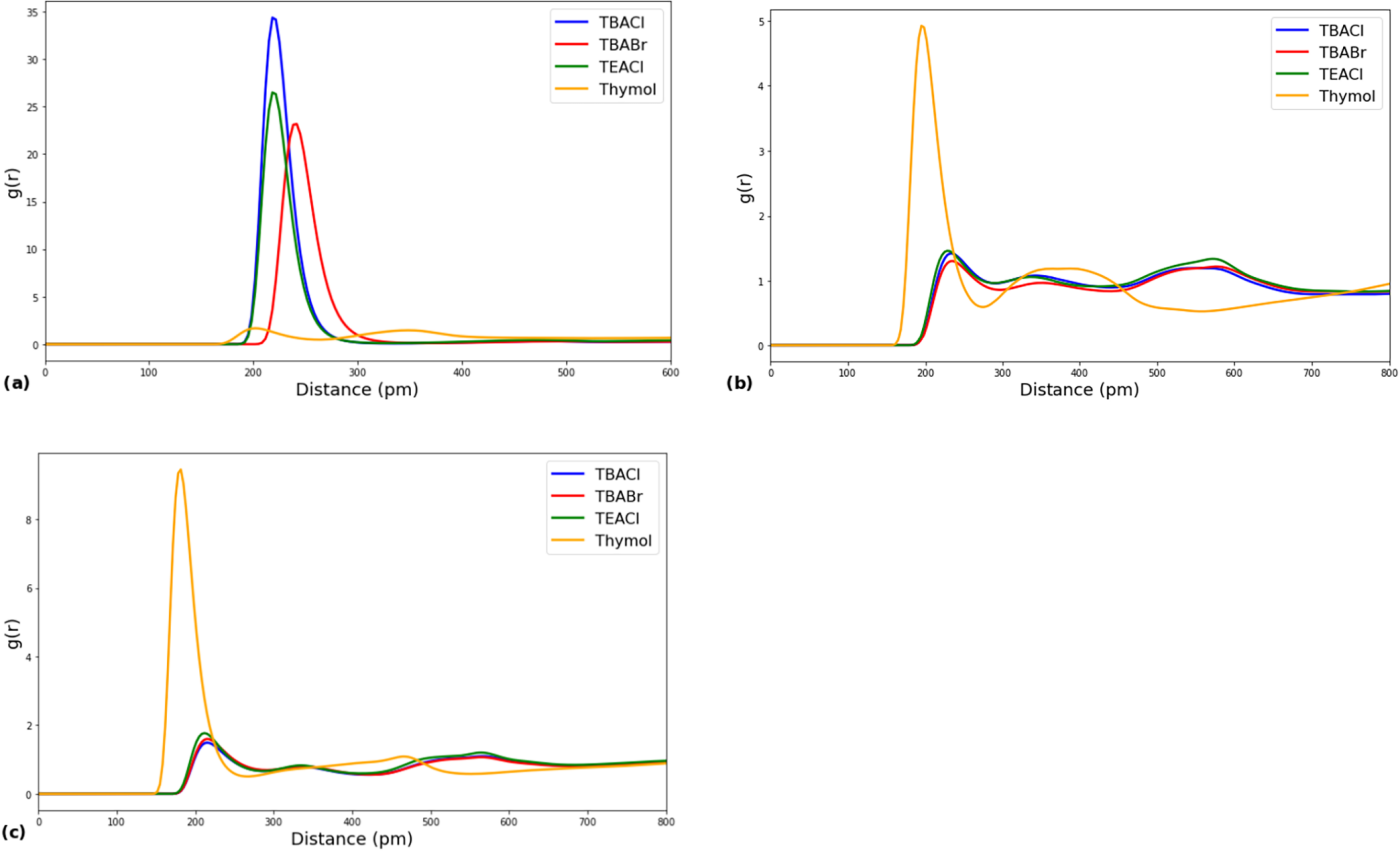
Atom specific RDFs that show hydrogen bonding interactions in 1:2 TBABr:EM (red), TBACl:EM (blue), TEACl:EM (green) and thymol:EM (orange) DES for (a) EM H9-anion or EM H9-thymol OT, (b) H_inner_-O2 (EM) and H5 (thymol)-O2, and (c) H_inner_-O3 (EM) and H5 (thymol)-O3. See Supporting Information (Figure S16) for atom numbering.

Table 3 shows the peak maxima and the coordination numbers determined from the RDFs in Figure 5. Table 3 also shows the % time bonded determined from an analysis of the hydrogen bond dynamics. This is the amount of time that the hydrogen bond distance criterion is met over the course of the simulation.^10,47,69,70^ The H9–Cl radii and coordination numbers are equivalent and there is a slightly smaller hydrogen bond persistence (% time bonded) for the TEACl vs TBACl DES. The H9–Br radius is longer (∼20 pm) and less persistent (∼3%) than the H9–Cl indicating weaker hydrogen bonding with the bromide. These results are consistent with the hydrogen bond acidity, α, and basicity, β, for chloride vs bromide DES shown in Table 2 which shows that the weaker hydrogen bonds with bromide can lead to stronger hydrogen bonding of the ethylmandelate with a solute in TBABr:EM DES.

**Table 3:** Hydrogen Bond Length, Coordination Number, and Persistence in Four DES

	TBACl			TBABr		
	*r*_max_ (pm)	N_coord_	% time bonded^a^	*r*_max_ (pm)	N_coord_	% time bonded
H9-anion	218	0.68	98.1	242	0.65	95.9
Hinner-O2	235	0.5	59.8	235	0.58	59.3
Hinner-O3	215	0.54	70.7	215	0.56	68.7

	TEACl			Thymol		
	*r*_max_ (pm)	N_coord_	% time bonded	*r*_max_ (pm)	N_coord_	% time bonded
H9-anion (H9-thymol OT)^b^	215	0.68	97.3	202	0.09	24.2
Hinner-O2 (H5–O2)^b^	225	0.73	48.7	198	0.16	55.4
Hinner-O3 (H5–O3)^b^	212	0.7	56.4	182	0.22	66.3

a The hydrogen bond lifetimes used to derive these values are in Table S9.

b The labels of the hydrogen bonds in thymol:EM are in parentheses.

The radii, coordination numbers and hydrogen bonding persistence are very similar for the cation-EM, Hinner-O2 and Hinner-O3, in TBACl vs TBABr (Table 3), where both show a preference in % time for the carbonyl oxygen (O3) of the ethyl mandelate. While these hydrogen bond radii are shorter and the coordination number is larger for TEA^+^ vs TBA^+^, the persistence of the Hinner-O2 and Hinner-O3 is shorter. The hydrogen bond distance and coordination numbers indicate a stronger hydrogen bond in TEACl:EM, which is consistent with this DES having the lowest α and β in Table 2. Conversely, TEACl:EM also has the shortest % time hydrogen bonded, which is inconsistent with the Kamlet–Taft parameters. It does seem clear that the hydrogen bonding of the cation-EM is important to the differences in solvation behavior of TBA-TEA, there is a complex interplay between hydrogen bond strength and dynamics.

The H9-OT (Figure 5 and Table 3) in thymol:EM shows a very small *g*(*r*), coordination number and hydrogen bond persistence that is consistent with previous observations that thymol is not a very good hydrogen bond acceptor.^7,85,86^ Both of the hydrogen bonds where thymol is the HBD, H5–O2 and H5–O3, show shorter radii, slightly higher coordination numbers, and much higher hydrogen bond persistence than the H9-OT. The data shown in Figure 5c and Table 3 shows that there is a higher prevalence of hydrogen bonding to the carbonyl oxygen (O3) vs the hydroxyl oxygen (O2) of EM, which is consistent with the hydrogen bond basicity demonstrated by EM in the ionic DES. Considering the probabilities for all of the possible thymol hydrogen bonding interactions in Figure 5 and Table 3, it is clear that thymol is functioning primarily as an HBD, not HBA.

### CPL Spectra

Figure 6 shows the CPL and average luminescence spectra for the ^5^D_0_ → ^7^F_0–2_ transitions of Eu­(dpa)_3_^3–^ dissolved in 1:2 TBACl: (R)- vs (S)-EM. The average luminescence shows the typical spectral pattern for Eu­(dpa)_3_^3–^ with a large peak at 615 nm for the ^5^D_0_ → ^7^F_2_ transition, two smaller peaks at 592 and 594 nm for the ^5^D_0_ → ^7^F_1_ transition, and an extremely weak ^5^D_0_ → ^7^F_0_ (symmetry forbidden) transition at 580 nm. Along with the long luminescence lifetimes (Table S5), the spectrum signifies that the structure of Eu­(dpa)_3_^3–^ is not changing when dissolved in the DES. The CPL spectra show peaks in 1:2 TBACl: (R)-EM (or (S)-EM) that are opposite sign for the ^5^D_0_ → ^7^F_2_ vs ^5^D_0_ → ^7^F_1_ transitions. This is a clear sign that racemic mixtures of Eu­(dpa)_3_^3–^ complexes has been enantiomerically resolved by the chiral DES. The CPL spectrum also shows peaks that are opposite sign in (R)- vs (S)-EM which shows that the enantioselectivity is controlled by the handedness the DES. The CPL spectra of Eu­(dpa)_3_^3–^ dissolved in the other five pairs of ionic (R)/(S) DES show similar spectral structure and sign based on (R)- vs (S)-EM/MM (shown in Figure S10). The Eu­(dpa)_3_^3–^ complexes would not dissolve in any of the DES with nonionic HBAs, thymol or 1,8-cineole.

**Figure 6:**
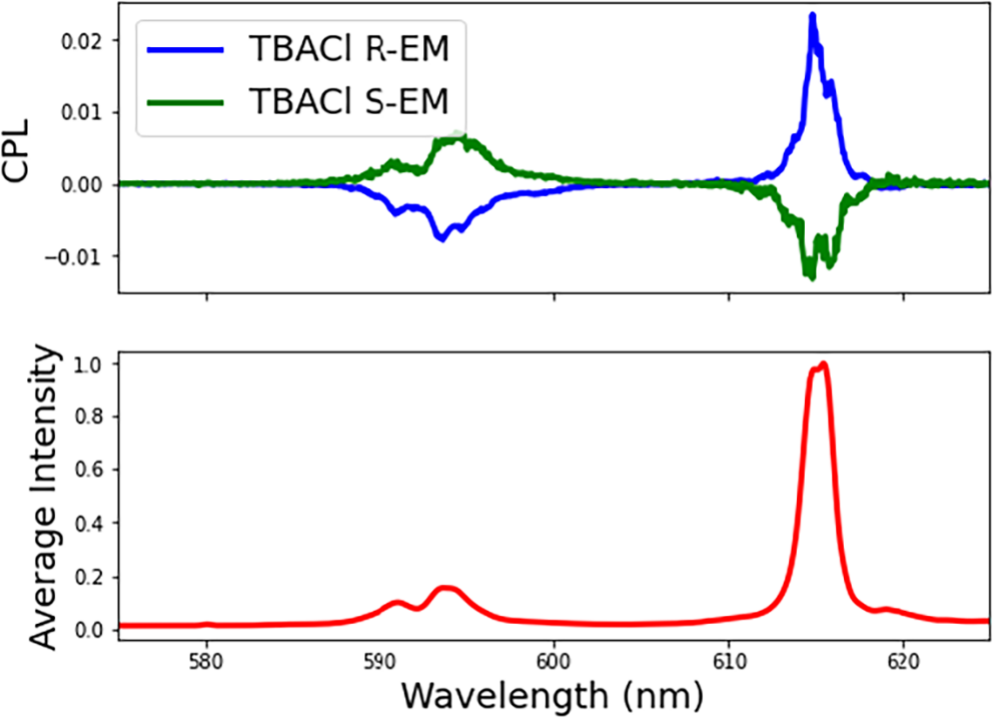
CPL spectrum of the ^5^D_0_ → ^7^F_0–2_ transitions of Eu­(dpa)_3_^3–^ dissolved in 1:2 TBACl: (S)-EM (green) vs 1:2 TBACl: (R)-EM (blue). The average luminescence spectra (red) are shown in the bottom frame.

### Enantioselectivity

The emission dissymmetry factor, *g*_em_(λ), is a measure of the degree of polarization of the emitted light given in eq 3.

3 $$g_{\mathrm{em}}\left(\lambda \right)=\frac{I_{L}-I_{R}}{\left(I_{L}+I_{R}\right)/2}$$

In the case of Eu­(dpa)_3_^3–^, the *g*_em_(λ) provides a measure of the shift in the racemization equilibrium, or the enantioselectivity, demonstrated by the chiral DES.^35^ Table 4 shows the measured *g*_em_(594 nm) in the ^5^D_0_ → ^7^F_1_ transition region for Eu­(dpa)_3_^3–^ dissolved in 12 ionic chiral DES. As is shown in Figure 6 and Table 4, the DES with (R)-EM or -MM have *g*_em_ < 0, while DES with (S)-EM or -MM have *g*_em_ > 0. Therefore, the enantiomer of the mandelate ester dictates the sign. Additionally, there is not a significant difference in the magnitudes of *g*_em_ for EM vs MM DES with a common HBA. The largest g_em_ in the table is attributed to TBABr: EM and MM, which corresponds to the DES with the largest hydrogen bond acidity, α (Table 2). Since the only hydrogen bond donor is on the chiral carbon of EM and MM, these DES would have the strongest interaction between the chiral HBD and the Eu­(dpa)_3_^3–^. The rest of the data in Table 4 shows that the alkyl chain length of the achiral HBA has an impact on the *g*_em_. However, the impact is not strictly based on the trend in HBA size, because the magnitude of *g*_em_ follows TBACl > TEACl > TPACl.

**Table 4:** Emission Dissymmetry Factors for Eu­(dpa)_3_^3–^ Dissolved in 1:2 Molar Ratio DES

DES	*g*_em_ (594 nm)^a^	DES	*g*_em_(594 nm)
TBABr:(R)-EM	–0.058	TBACl:(R)-EM	–0.044
TBABr:(S)-EM	+0.066	TBACl:(S)-EM	+0.037
TPACl:(R)-EM	–0.025	TEACl:(R)-EM	–0.038
TPACl:(S)-EM	+0.025	TEACl:(S)-EM	+0.033
TBABr:(R)-MM	–0.079	TBACl:(R)-MM	–0.044
TBABr:(S)-MM	+0.083	TBACl:(S)-MM	+0.045

a Uncertainty = ±0.002.

The *g*_em_ measured in Table 4 arise from a chiral solvation interaction that perturbs the racemization equilibrium of Eu­(dpa)_3_^3–^

4 $$\Delta ‐Eu(dpa{)}_{3}^{3-}⇌\Lambda ‐Eu(dpa{)}_{3}^{3-},K_{\mathrm{rac}}$$

where *K*_rac_ is the equilibrium constant. The conversion of *g*_em_(594) to *K*_rac_ has been described in detail previously.^35,52^ Assuming that the dissymmetry factor for an enantiomerically resolved population of Λ-Eu­(dpa)_3_^3–^ is *g*_em_^Λ^ = 0.29,^52^ the equilibrium constant can be determined by

5 $$K_{\mathrm{rac}}=\frac{1+\frac{g_{\mathrm{em}}}{g_{\mathrm{em}}^{\Lambda }}}{1-\frac{g_{\mathrm{em}}}{g_{\mathrm{em}}^{\Lambda }}}$$

Where eq 5 can be used to convert *g*_em_ measurements into equilibrium constants, and their temperature dependence can be analyzed using the van’t Hoff equation

6 $$\ln K_{\mathrm{rac}}=-\frac{\Delta H_{\mathrm{rac}}}{\mathrm{RT}}+\frac{\Delta S_{\mathrm{rac}}}{R}$$

where *R* is the gas constant, Δ*H*_rac_ and Δ*S*_rac_ are the enthalpy and entropy of the racemization equilibrium. The Δ*H*_rac_ quantifies the strength of the enantioselective interactions between the chiral DES and Eu­(dpa)_3_^3–^ (similar to a ΔΔ*H*). The Δ*S*_rac_ relates to the differential orientation of the chiral DES solvent about the Λ- vs Δ-enantiomers (a ΔΔ*S*).

Table 5 shows the results from the van’t Hoff analysis (shown in Figures S11–S15), Δ*H*_rac_ and Δ*S*_rac_, along with the Δ*G*_rac_ at 298 K for 10 chiral ionic DES. Samples in 1:2 TPACl: (R)- and (S)-EM did not fit eq 5, because the *g*_em_ got vanishingly small at higher temperatures. Even though 1:2 TPACl: (R)/(S)-EM had the lowest *g*_em_ in Table 4, it is unclear why there is a significant departure in the temperature dependence vs the other EM DES. The data in Table 5 shows that all DES with (R)-EM or MM as the chiral HBD shift the equilibrium toward Λ-Eu­(dpa)_3_^3–^, where the (S)-EM and -MM shift the equilibrium to the Δ*e*nantiomer. For all DES in Table 5, the enthalpy favors the observed enantioselectivity indicating that the solvent–solute intermolecular interactions, such as hydrogen bonding, are enantioselective. The entropy for all DES opposes the observed enantioselectivity, which shows that any enantioselective ordering by the chiral DES the inherent entropic advantage to being racemic over enantiomerically resolved. The Br^–^ DES show the largest enthalpies, |Δ*H*_rac_| ∼ 2–4 kJ/mol, and |Δ*G*_rac_| ∼ 1–2 kJ/mol, while the Cl^–^ DES have identical free energies of enantioselectivity, |Δ*G*_rac_| = 700 J/mol. This is consistent with the larger hydrogen bond acidity, α, exhibited by the Br^–^ DES. The simulation results also show weaker H9–Br^–^ hydrogen bonding influencing the enantioselectivity because the O2–H9 is bonded to the chiral carbon center in EM. For reference, the thermodynamic parameters for the chloride DES in Table 5 are similar in magnitude to and the bromide DES are larger than those determined for amino acid–based ionic liquids and amino acid DES.^35,52^ To provide a comparison, the enantioselectivity of β-cyclodextrin in two different studies gives |ΔΔ*H*| = 0.5–7 kJ/mol^87^ and |ΔΔ*G*| = 0.1–0.4 kJ/mol,^88^ which means that the chiral EM and MM DES have potential as a chiral separation media.

**Table 5:** Results from the van’t Hoff Analysis of the Racemization Equilibrium in Chiral DES

DES^a^	Δ*H*_rac_ (kJ/mol)	Δ*S*_rac_ (J/K·mol)	Δ*G*_rac_ (kJ/mol) at 298 K
TBABr:(R)-EM	4.4	9.1	1.7
TBABr:(S)-EM	–3.2	–7.9	–0.8
TBACl:(R)-EM	1.8	3.5	0.7
TBACl:(S)-EM	–2.6	–6.6	–0.6
TEACl:(R)-EM	1.9	4.1	0.6
TEACl:(S)-EM	–1.6	–3.3	–0.6
TBACl:(R)-MM	2.5	5.8	0.7
TBACl:(S)-MM	–1.5	–2.6	–0.7
TBABr:(R)-MM	2.6	4.2	1.3
TBABr:(S)-MM	–2.3	–3.2	–1.4

a All DES are 1:2 molar ratio.

### Why EM over MM?

In a previous study, EM mixtures formed liquids at 20 °C with each of the HBAs attempted, whereas MM formed liquids with fewer HBAs at fewer molar ratios.^39^ This is one reason that this study characterized the properties of 12 DES with R- or S-EM versus only four DES with R- or S-MM. Comparison of the simulation results for 1:2 TBACl:(R)-EM vs (R)-MM does lead to insight into the difference between the two HBAs. Figure 7 shows the dihedral distribution functions of the dihedral angles associated with the ester in MM vs EM. The four atoms in the methyl ester (MM) and the same four atoms of the ethyl ester have dihedral angles narrowly centered around 0° (Figure 7a). However, the four atoms that include both carbons of the ethyl ester have multiple conformers with dihedral angles, ±80° and 180° as shown (Figure 7b). This shows that the ethyl ester of EM provides conformational flexibility that could frustrate crystallization in EM. This could help explain why EM is more effective as an HBD in DES formation than MM.

**Figure 7:**
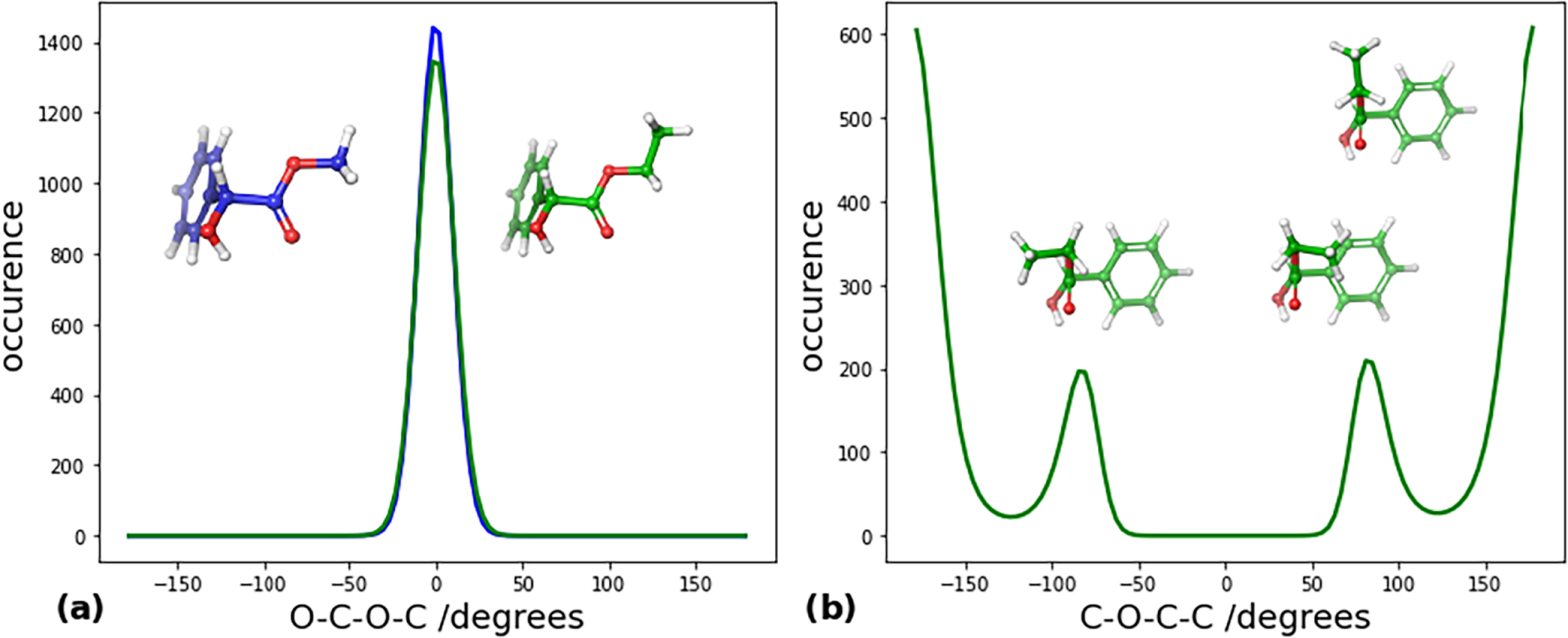
Dihedral distribution functions for (a) the dihedral angle formed by O3–C2–O1–C9 of methyl mandelate (blue) in 1:2 TBACl:MM and the interior dihedral, O3–C2–O1–C4, of ethyl mandelate (green) in 1:2 TBACl:EM, and (b) the exterior dihedral, C2–O1–C4–C10, of ethyl mandelate (green) in 1:2 TBACl:EM. Inset in (b) are stick model representations showing the conformers of EM for each of the dihedral angles. The atom numbering is included in Supporting Information (Figure S16).

## Conclusion

In this study, ethyl and methyl esters of mandelic acid are chiral HBDs in the formation of chiral DES/eutectic mixtures with tetraalkylammonium salts as well as nonionic HBAs, thymol, and 1,8-cineole. DSC measurements indicate that the eutectic point occurs at 1:2 TBABr:EM and 2:1 thymol:EM. Thermodynamic analysis of the solid–liquid equilibrium curve shows that EM forms a DES in both ionic and nonionic mixtures. Measurements of density and viscosity of 1:2 HBA:(R)- vs (S)-EM/MM show very little difference based on the enantiomer of the HBD. The Kamlet–Taft parameters show that the hydrogen bond acidity of Br^–^ is greater than in Cl^–^ eutectic mixtures due to weaker HBD-Br hydrogen bonds. The enantioselectivity of the ionic chiral EM DES was measured by their ability to induce CPL in a racemic mixture of luminescent lanthanide complexes. These measurements show an increase in enantioselectivity for Br^–^ vs Cl^–^ DES, which is the result of the increase in hydrogen bond acidity of EM in bromide eutectic mixtures. Thermodynamic parameters measured for the chiral DES show enantioselectivities that can be used in chiral selection applications. MD simulation results confirm that hydrogen bonding of the EM with both cation and anion of the ionic HBA are important, and that thymol functions more as an HBD than HBA. Simulation data shows that EM has conformational flexibility in the ester that could help explain why EM is more effective in forming DES/eutectic mixtures. Ethyl mandelate forms chiral DES/eutectic mixtures with a structurally diverse set of HBAs exhibiting substantial enantioselectivity.

## Supplementary Material



## Notes

Water content and mole fractions for all mixtures, DSC traces for melting of (R)- vs (S)-EM, mole fractions of TBABr:EM, thymol:EM, and cineole:EM, DSC traces for all DES, solid–liquid equilibrium curve for 1,8-cineole:EM, densities, conductivities, viscosities and Kamlet–Taft parameters for all of the DES, luminescence lifetimes of Eu­(dpa)_3_^3–^ in the DES, CPL spectra and van’t Hoff analysis for all DES, atom numbering for molecular structures, COM and RDFs for four DES, NAMD stream files to define topology and supply missing force field parameters (PDF)
