# Supplementary material for: Esters of Mandelic Acid as a Basis for Chiral Eutectic Solvents: An Investigation of Physical Properties and Enantioselectivity
Source: J Phys Chem B. 2026 May 22;130(22):5588–600. doi: 10.1021/acs.jpcb.5c05034 (PMC13244473; doi:10.1021/acs.jpcb.5c05034)
Supplement: Supplementary file 1 [file jp5c05034_si_001.pdf]

Supporting Information for

Esters of Mandelic Acid as a basis for Chiral Eutectic Solvents: An Investigation of Physical Properties and Enantioselectivity

Olivia A. Gooch, Jenna N. Lane, Mandy Bechman, Valentine J. Klimkowski, Todd A. Hopkins

Department of Chemistry and Biochemistry, Butler University, 4600 Sunset Avenue, Indianapolis, IN 46208.

Table of Contents

1. Table of mole fractions for each mixture
2. DSC heating-cooling curve for 1:1 TBABr: (R)-EM. DSC heating curves (R)- and (S)-EM, and all mixtures of TBABr:EM, thymol:EM, and 1,8-cineole:EM.
3. Solid-liquid equilibrium curve for 1,8-cineole:EM
4. DSC curves for all DES near the eutectic point
5. Thermodynamics of fusion properties for pure components
6. Table with density and viscosity for the mixtures, and temperature-dependent viscosity/conductivity plots for many of the EM/MM mixtures.
7. Kamlet-Taft parameters measured for R- vs. S- DES.
8. Luminescence lifetimes for $Eu(dpa)_3^{3-}$ dissolved in the mixtures
9. CPL spectra of $Eu(dpa)_3^{3-}$ in all of the DES
10. Example of T-dependent CPL and Van't Hoff plot.
11. Component compositions for the simulations
12. Atom numbering for the components of the simulations
13. Center of mass and atom specific RDFs for the reference molecules and atoms not shown in main text.
14. NAMD stream files

Table S1. Component mole fractions with water in eutectic mixtures.

Mixture	x_{HBA}[a]	x_{EM}	x_w
1:2 TBABr: (R)-EM	0.319	0.639	0.041
1:2 TBABr: (S)-EM	0.327	0.654	0.019
1:2 TBACl: (R)-EM	0.317	0.633	0.050
1:2 TBACl: (S)-EM	0.261<br>(0.332)	0.522<br>(0.665)	0.218<br>(0.002)
1:2 TPACl: (R)-EM	0.269	0.538	0.193

	(0.331)	(0.662)	(0.006)
1:2 TPACl: (S)-EM	0.251<br>(0.326)	0.503<br>(0.652)	0.246<br>(0.021)
1:2 TEACl: (R)-EM	0.267<br>(0.318)	0.534<br>(0.636)	0.198<br>(0.047)
1:2 TEACl: (S)-EM	0.265<br>(0.330)	0.530<br>(0.660)	0.204<br>(0.010)
1:2 Thymol: (R)-EM	0.323	0.646	0.030
1:2 Thymol: (S)-EM	0.324	0.648	0.028
1:2 Cineole: (R)-EM	0.314	0.628	0.059
1:2 Cineole: (S)-EM	0.321	0.642	0.038
1:2 TBACl: (R)-MM	0.306	0.611	0.083
1:2 TBACl: (S)-MM	0.277<br>(0.326)	0.555<br>(0.652)	0.167<br>(0.022)
1:2 TBABr: (R)-MM	0.328	0.656	0.016
1:2 TBABr: (S)-MM	0.321	0.641	0.038

[a] mole fractions in parentheses have undergone additional vacuum drying. All other mole fractions for samples prepared and stored in ambient conditions.

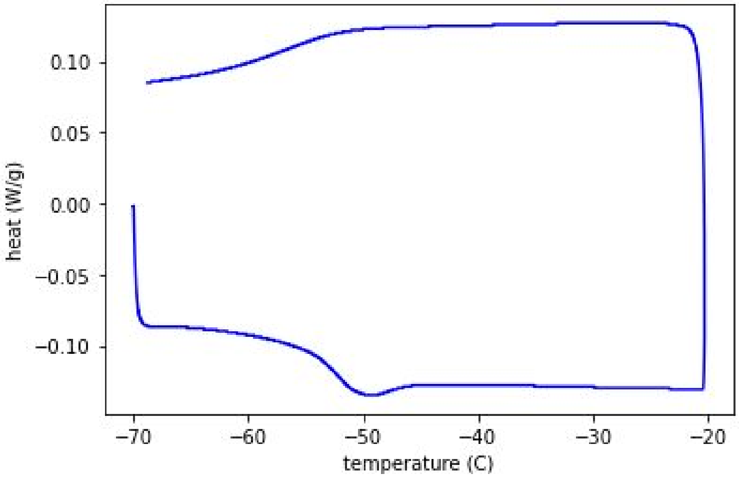

Figure S1. DSC heating-cooling curve of 1:1 TBABr: (S)-EM.

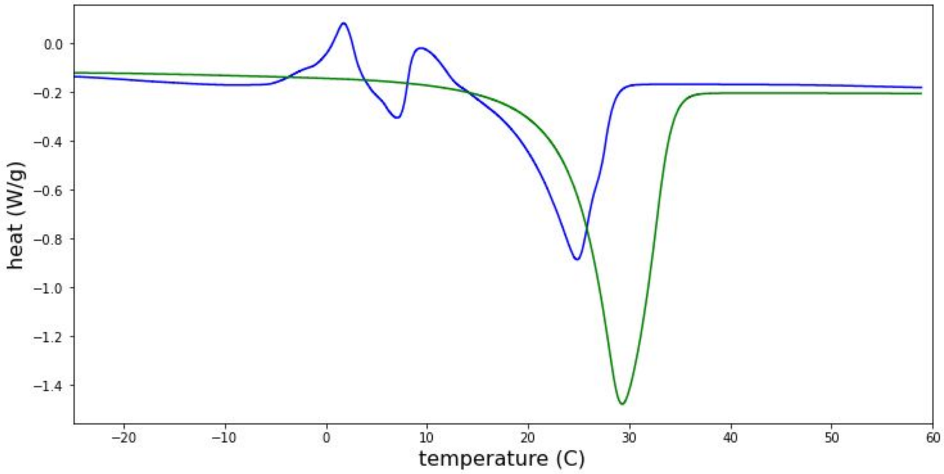

Figure S2. DSC thermograms of (R)-EM (blue) vs. (S)-EM (green).

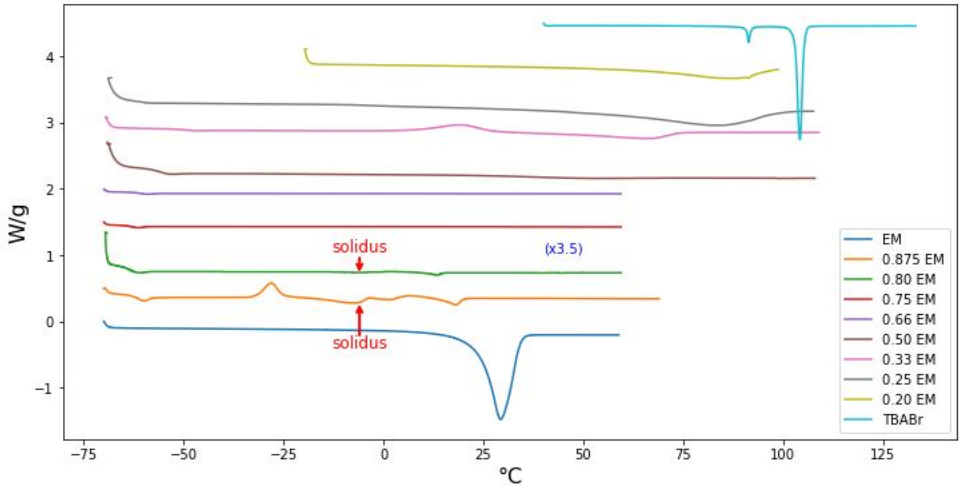

Figure S3. DSC thermograms for mixtures of TBABr with (S)-EM. The x_{EM} = 0.80 thermogram has small endothermic peaks at 13 °C and -6 °C which do not show up on the scale of the graph. The red arrows point to the experimentally observed solidus transition.

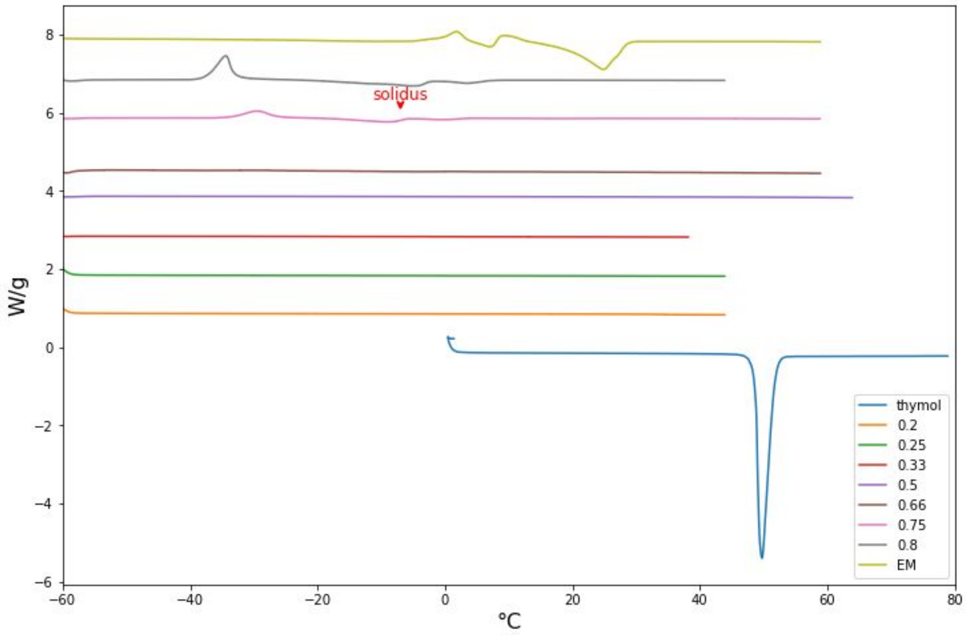

Figure S4. DSC thermograms for mixtures of thymol with (S)-EM. The x_{EM} = 0.33 thermogram has a small endothermic peak at -5 °C that does not show up on the scale of the graph. The red arrows point to the experimentally observed solidus transition.

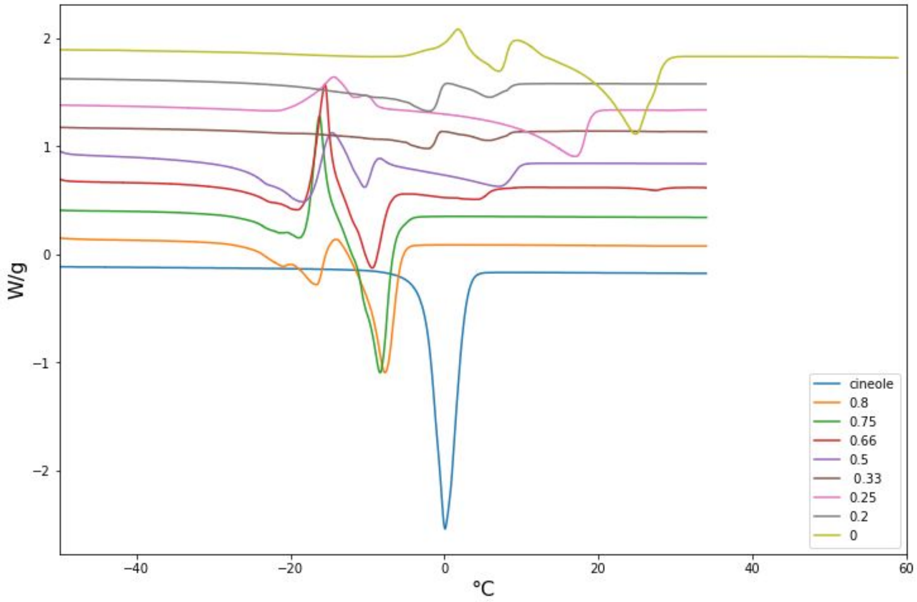

Figure S5. DSC thermograms for mixtures of 1,8-cineole with (S)-EM.

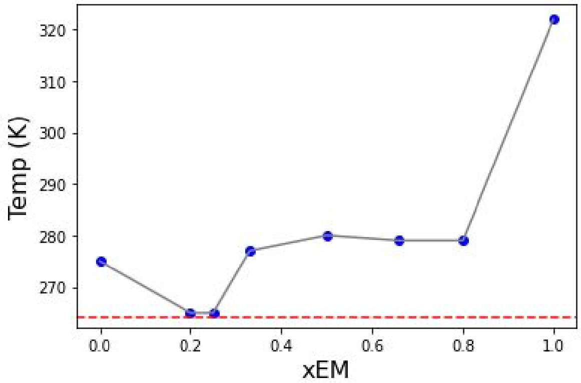

Figure S6. Solid-liquid equilibrium curve for 1,8-cineole mixed with (S)-EM. The dotted red line shows the solidus line, where the solid blue line represents the liquidus line.

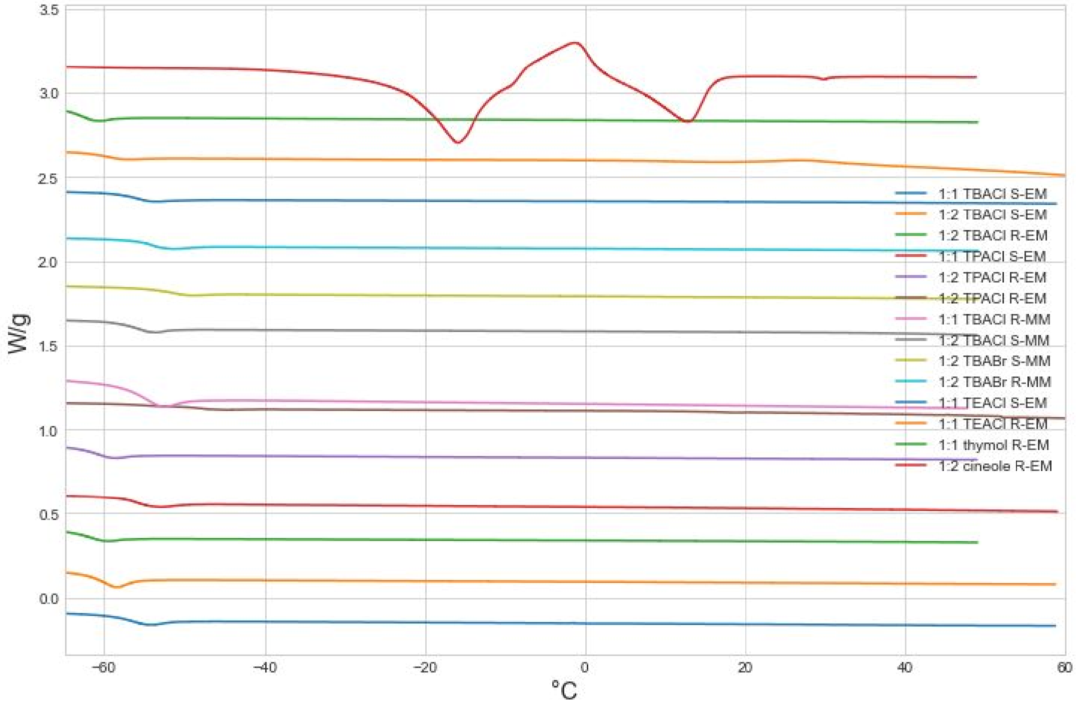

Figure S7. DSC plots for TBABr: (R)-EM, TBACl: (S)/(R)-EM, TPACl: (S)/(R)-EM, TBACl: (S)/(R)-MM, TBABr: (S)/(R)-MM, TEACl: (S)/(R)-EM, thymol: (R)-EM, and 1,8-cineole: (R)-EM

Table S2. Thermodynamic properties of the pure components.

	Melting point (°C)[a]	Enthalpy of fusion (kJ/mol)[a]
TBABr	104	16.15
Thymol	49	22.01
EM	29	20.88

[a] from ref NIST Webbook

Table S3. Densities and viscosities for DES at 293 K.

Mixture	Density (g/mL)	Viscosity (mPa s)
1:2 TBABr: (R)-EM	1.130	467
1:2 TBABr: (S)-EM	1.124	356
1:2 TBACl: (R)-EM	1.035	532
1:2 TBACl: (S)-EM	1.046	498
1:2 TPACl: (R)-EM	1.076	626
1:2 TPACl: (S)-EM	1.077	699
1:2 TEACl: (R)-EM	1.097	210
1:2 TEACl: (S)-EM	1.101	230
1:2 Thymol: (R)-EM	1.071	38
1:2 Thymol: (S)-EM	1.076	31
1:2 Cineole: (R)-EM	1.057	23
1:2 Cineole: (S)-EM	1.063	15

1:2 TBACl: (R)-MM	1.097	690
1:2 TBACl: (S)-MM	1.060	869
1:2 TBABr: (R)-MM	1.130	795
1:2 TBABr: (S)-MM	1.122	922

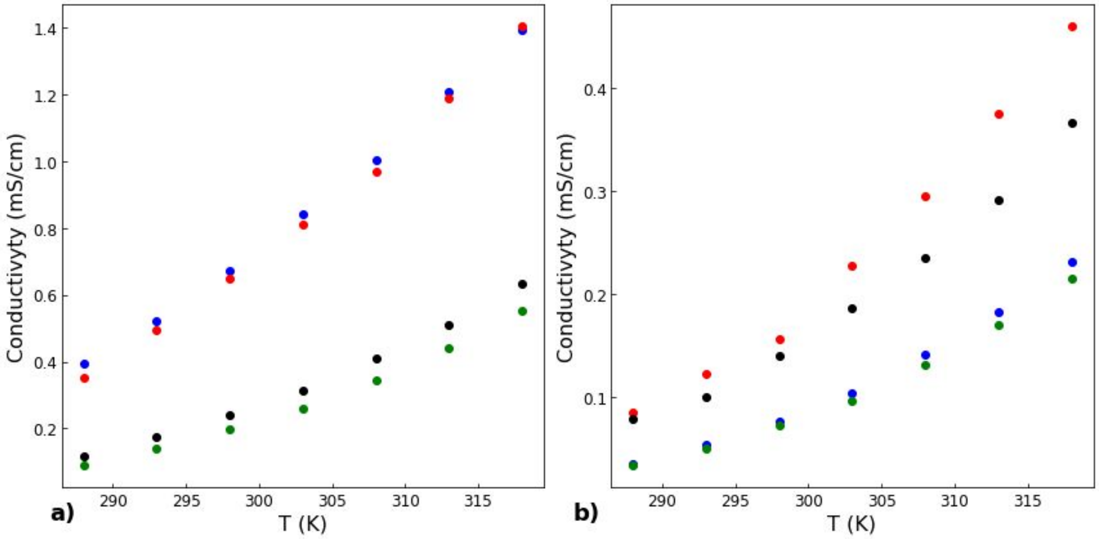

Figure S8. Conductivity vs. temperature for a) 1:2 TEACl: (R)-EM (blue), 1:2 TEACl: (S)-EM (red), 1:2 TPACl: (R)-EM (green), and 1:2 TPACl: (S)-EM (black) and b) 1:2 TBABr: (R)-EM (blue), 1:2 TBABr: (S)-EM (green), 1:2 TBACl: (R)-EM (black) and 1:2 TBACl: (S)-EM (red).

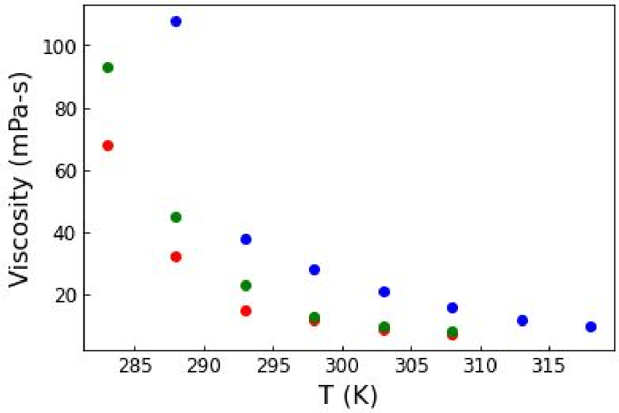

Figure S9. Viscosity vs. temperature for 1:2 thymol: (R)-EM (blue), 1:2 1,8-cineole: (R)-EM (green), and 1:2 1,8-cineole: (S)-EM (red).

Table S4. Kamlet-Taft parameters determined for DES

DES	α^{a}	β^{a}	π^{*a}
1:2 TBABr: (R)-EM	0.74	1.23	1.29
1:2 TBABr: (S)-EM	0.77	1.11	1.39
1:2 TBACl: (R)-EM	0.54	1.16	1.11
1:2 TBACl: (S)-EM	0.54	1.12	1.11
1:2 TPACl: (R)-EM	0.54	1.12	1.12
1:2 TPACl: (S)-EM	0.55	1.25	1.00

1:2 TEACl: (R)-EM	0.44	1.04	1.34
1:2 TEACl: (S)-EM	0.52	0.95	1.34
1:2 Thymol: (R)-EM	0.72	1.16	1.06
1:2 Thymol: (S)-EM	0.74	1.10	1.06
1:2 Cineole: (R)-EM	0.42	1.20	1.02
1:2 Cineole: (S)-EM	0.36	1.20	1.02
1:2 TBACl: (R)-MM	0.64	0.73	0.96
1:2 TBABr: (R)-MM	0.84	0.84	0.89

[a] uncertainty in α and $\beta = \pm 0.12$, and $\pi^* = \pm 0.06$.

Table S5. Luminescent lifetimes ($^5D_0$) of $Eu(dpa)_3^{3-}$ dissolved in 1:2 mole ratio DES.

DES	Lifetime (ms)[a]	DES	Lifetime (ms)
TBABr: (R)-EM	1.95	TBACl: (R)-EM	2.07
TBABr: (S)-EM	1.95	TBACl: (S)-EM	1.50
TPACl: (R)-EM	1.67	TEACl: (R)-EM	1.75
TPACl: (S)-EM	0.90	TEACl: (S)-EM	1.87
TBABr: (R)-MM	2.14	TBACl: (R)-MM	1.87
TBABr: (S)-MM	2.16	TBACl: (S)-MM	1.99

[a] uncertainty < ± 0.01 ms

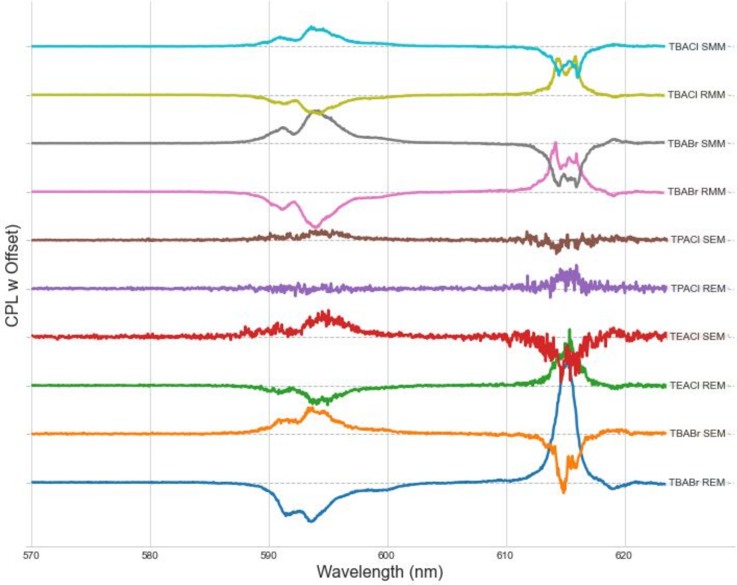

Figure S10. CPL spectra for 1:2 TBABr, TEACl, and TPACl with (R)/(S)-EM, 1:2 TBABr and TBACl with (R)/(S)-MM. The (R) spectra are negative and the (S) spectra are positive at 594 nm.

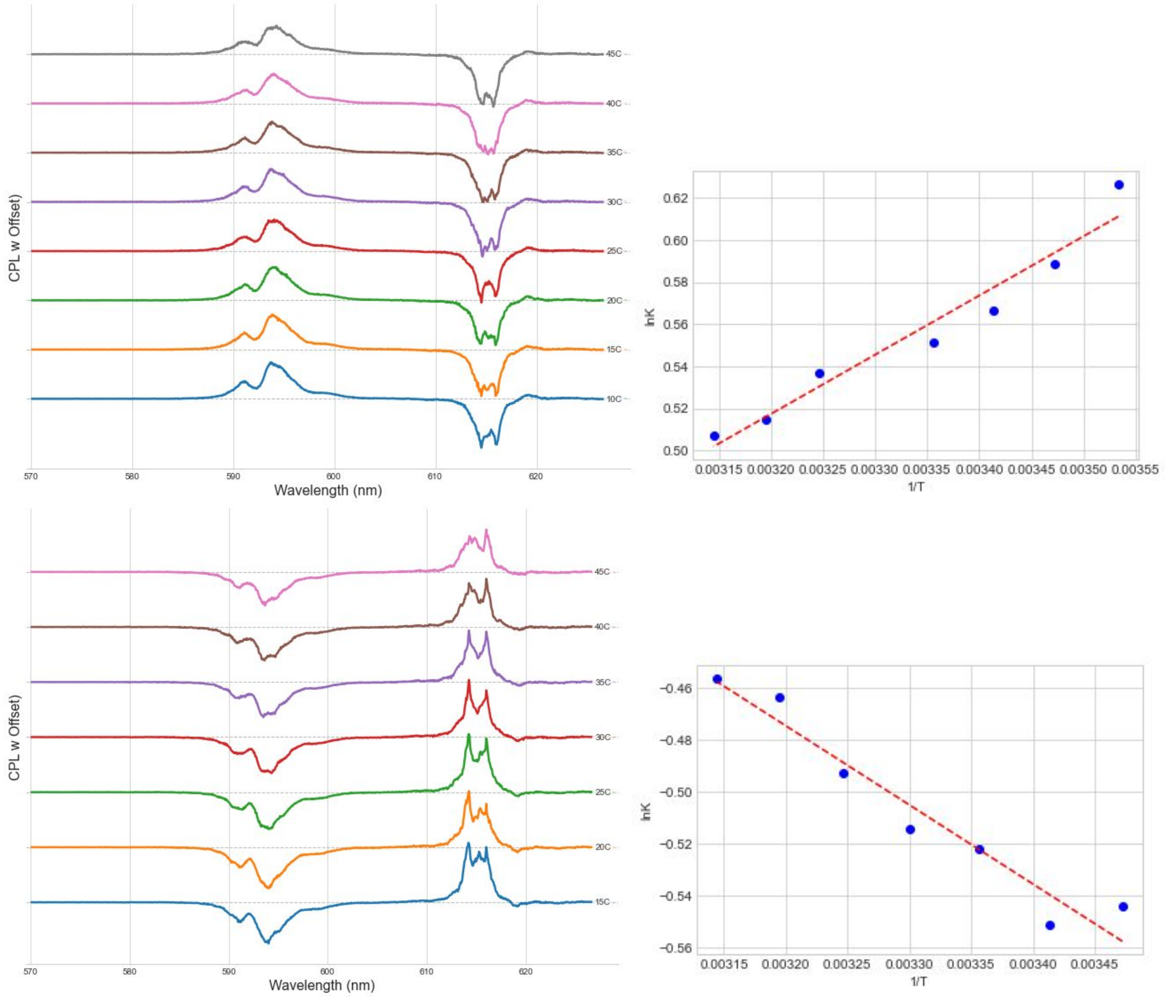

Figure S11. Temperature dependent CPL spectra (left panels) for $Eu(dpa)_3^{3-}$ in 1:2 TBABr: (S)- vs. (R)-MM, and the van't Hoff plot for the data (right panels).

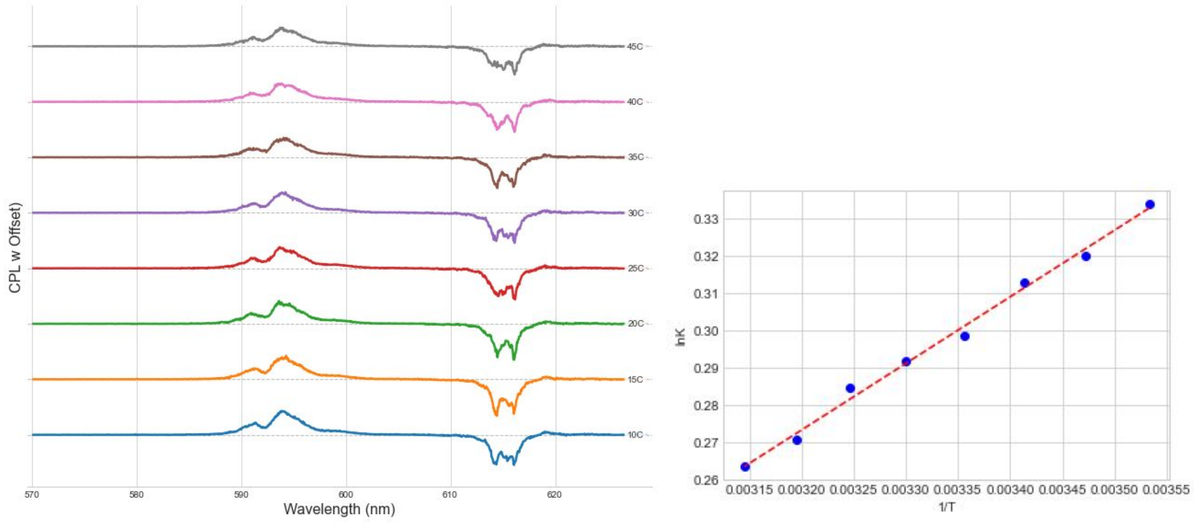

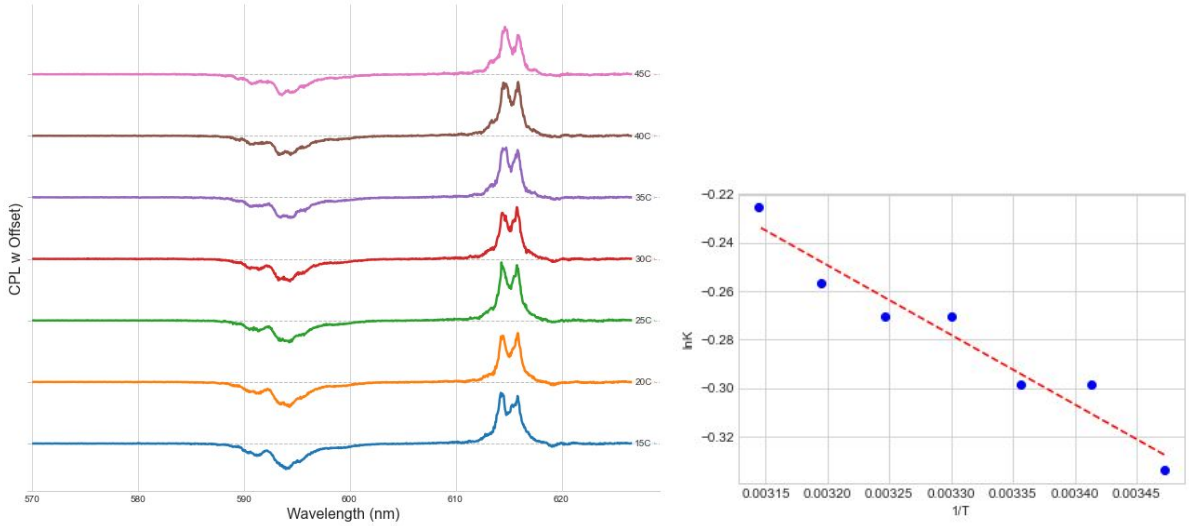

Figure S12. Temperature dependent CPL spectra (left panels) for $Eu(dpa)_3^{3-}$ in 1:2 TBACl: (S)- vs. (R)-MM, and the van't Hoff plot for the data (right panels).

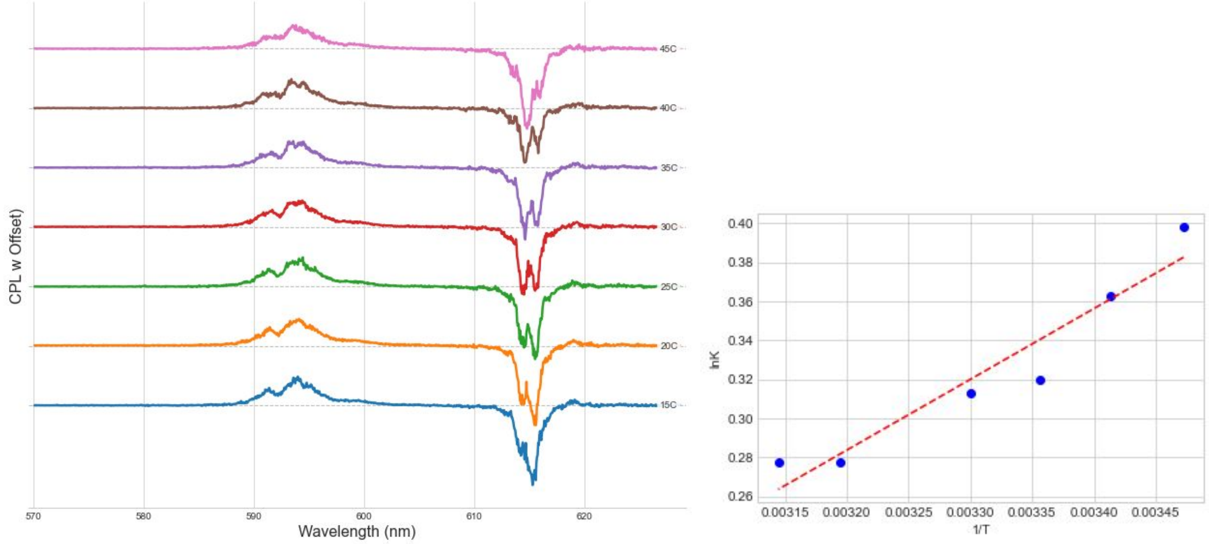

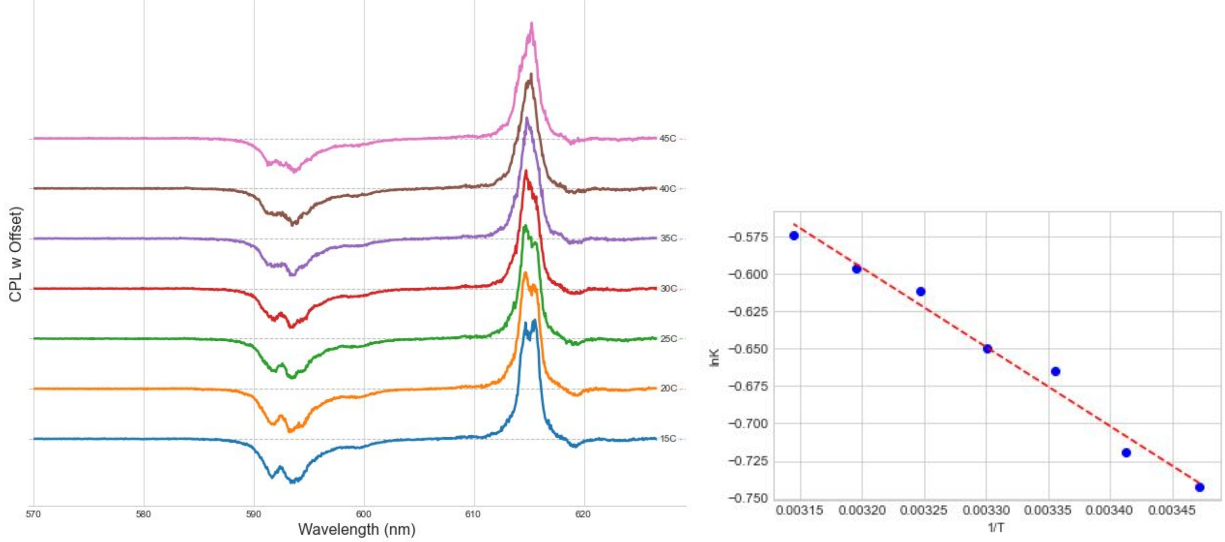

Figure S13. Temperature dependent CPL spectra (left panels) for $Eu(dpa)_3^{3-}$ in 1:2 TBABr: (S)- vs. (R)-EM, and the van't Hoff plot for the data (right panels).

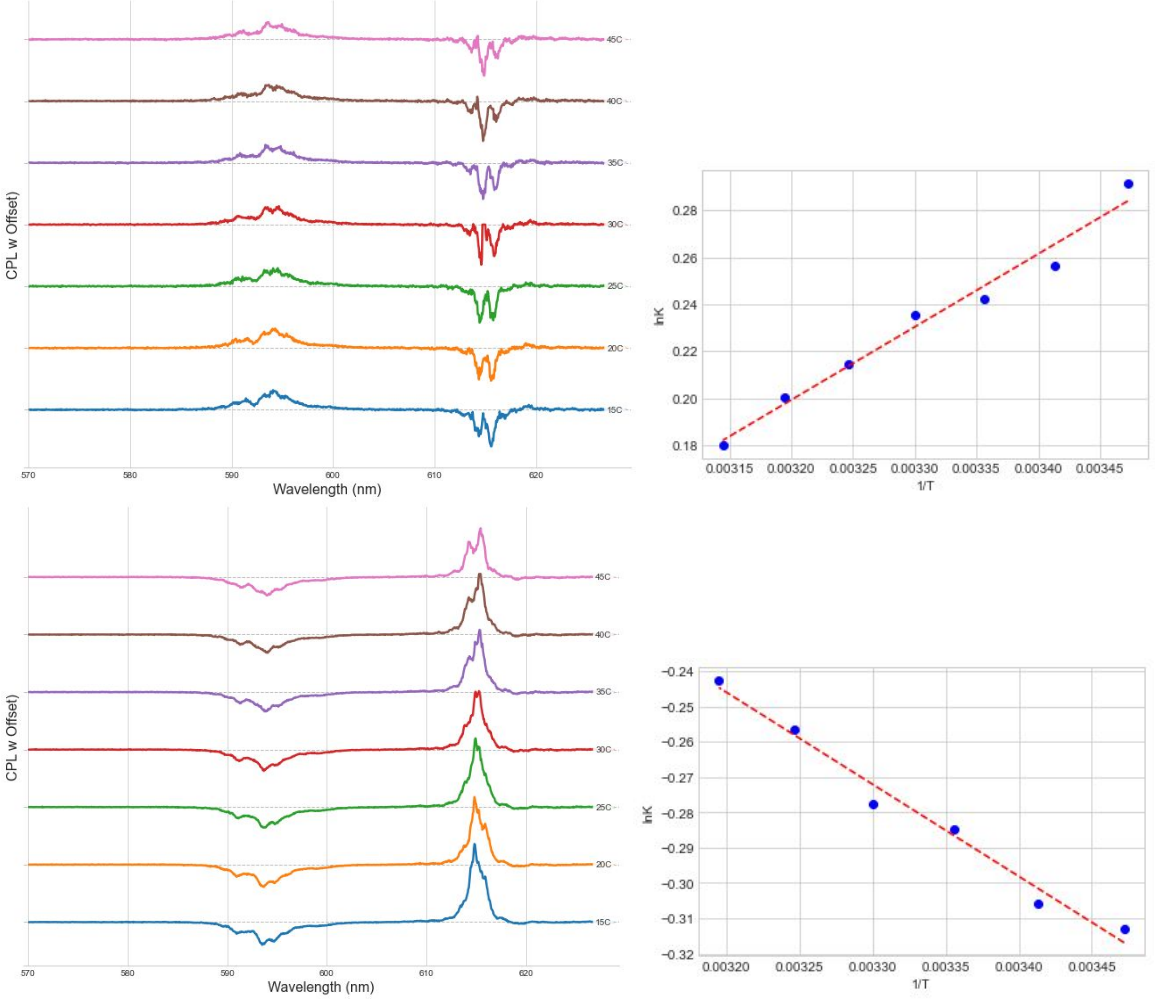

Figure S14. Temperature dependent CPL spectra (left panels) for $Eu(dpa)_3^{3-}$ in 1:2 TBACl: (S)- vs. (R)-EM, and the van't Hoff plot for the data (right panels).

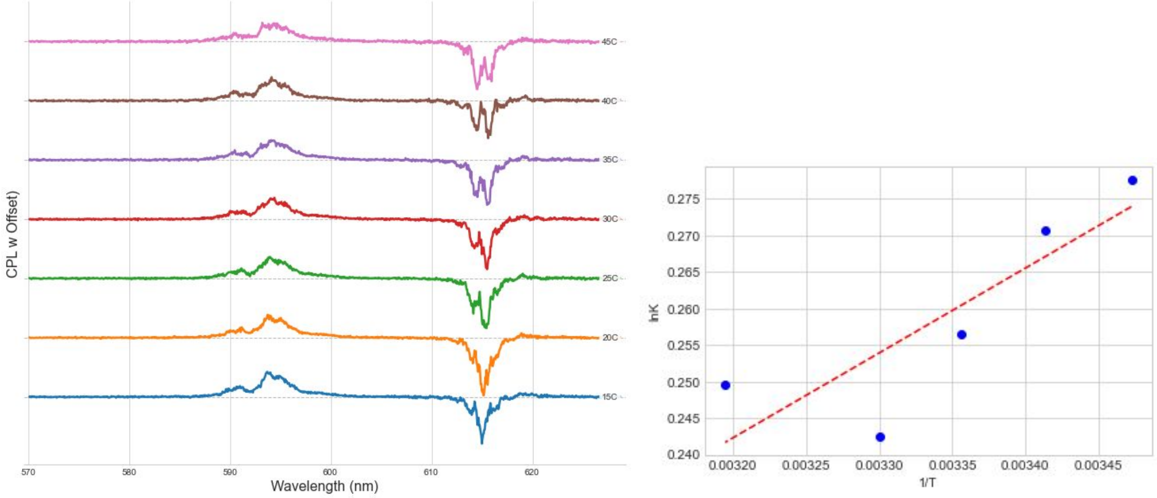

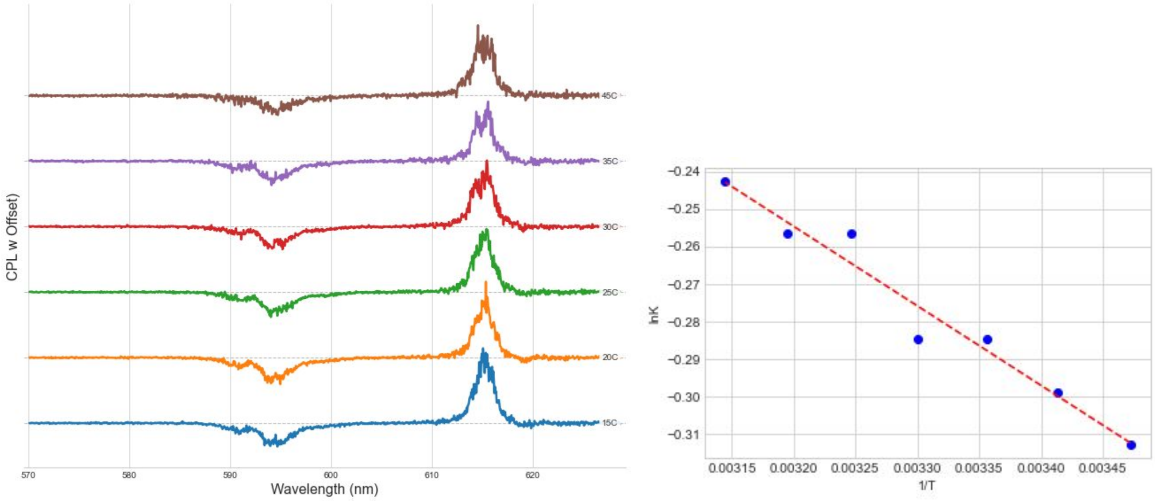

Figure S15. Temperature dependent CPL spectra (left panels) for $Eu(dpa)_3^{3-}$ in 1:2 TEACl: (S)- vs. (R)-EM, and the van't Hoff plot for the data (right panels).

Table S6. Component composition for simulated systems.

MD Run	Mixture	# Ion Pairs	# HBD	# waters	Eu Complex	Init Cube Length (Å)	Final Cube Length (Å)
1	1:2 TBABr: (R)-EM	100	200	10	Λ	48.40	47.82
2	1:2 TBABr: (R)-EM	100	200	10	Δ	48.40	47.81
3	1:2 TBABr: (S)-EM	100	200	10	Λ	48.40	47.84
4	1:2 TBABr: (S)-EM	100	200	10	Δ	48.40	47.85
5	1:2 TBACl: (R)-EM	100	200	16	Λ	47.50	47.52
6	1:2 TBACl: (R)-EM	100	200	16	Δ	47.50	47.57
7	1:2 TBACl: (S)-EM	100	200	77	Λ	47.50	47.66
8	1:2 TBACl: (S)-EM	100	200	77	Δ	47.50	47.67
9	1:2 TEACl: (R)-EM	100	200	76	Λ	44.00	44.12
10	1:2 TEACl: (R)-EM	100	200	76	Δ	44.00	44.11
11	1:2 TEACl: (S)-EM	100	200	76	Λ	44.00	44.10
12	1:2 TEACl: (S)-EM	100	200	76	Δ	44.00	44.13
13	1:2 Thymol: (R)-EM	100	200	3	none	43.95	43.39
14	1:2 Thymol: (S)-EM	100	200	3	none	43.95	43.38
15	1:2 TBACl: (R)-MM	100	200	45	none	46.83	46.75

Table S7. Comparison of Experimental vs. Simulated densities

Mixture	Exp. Density (g/mL)	MD Density (g/mL)	% difference
1:2 TEACl: (R)-EM	1.097	1.049	-4.48
1:2 TEACl: (S)-EM	1.101	1.051	-4.54
1:2 TBACl: (R)-EM	1.035	1.005	-2.90
1:2 TBACl: (S)-EM	1.046	1.005	-3.92
1:2 TBABr: (R)-EM	1.130	1.047	-7.34

1:2 TBABr: (S)-EM	1.124	1.045	-7.03
1:2 Thymol: (R)-EM	1.071	1.034	-3.45
1:2 Thymol: (S)-EM	1.076	1.037	-3.62

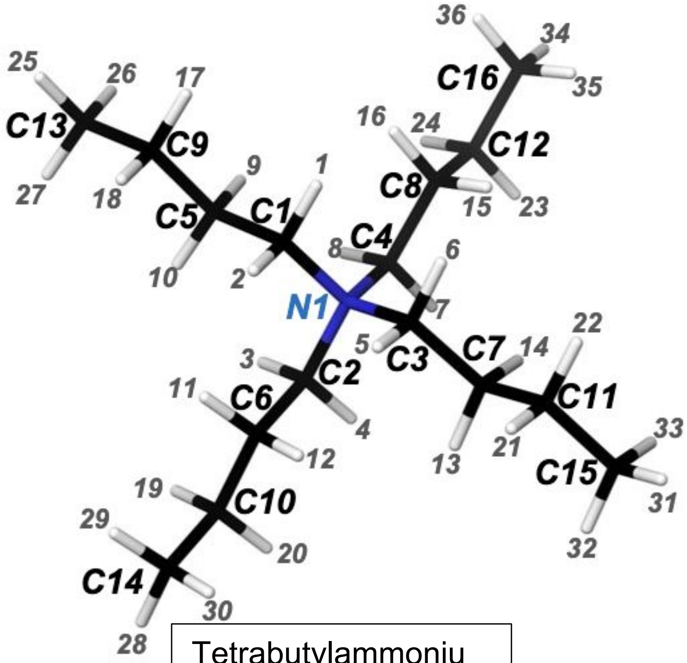

Tetrabutylammoniu

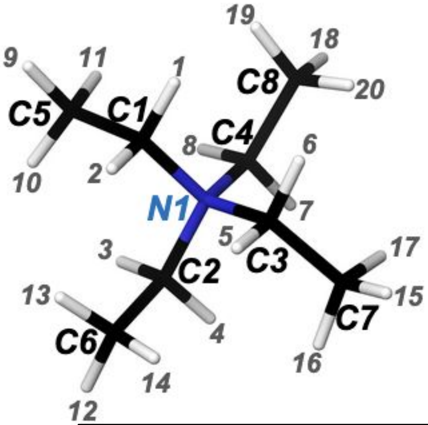

Tetraethylammonium

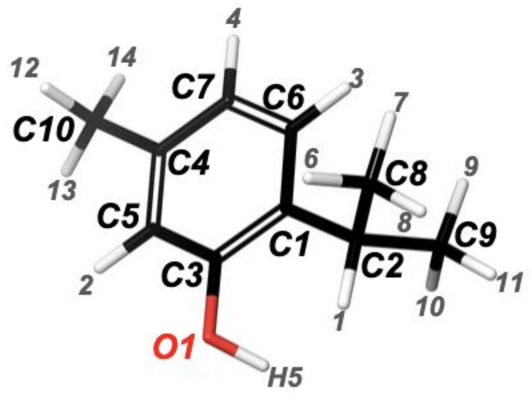

Thymol

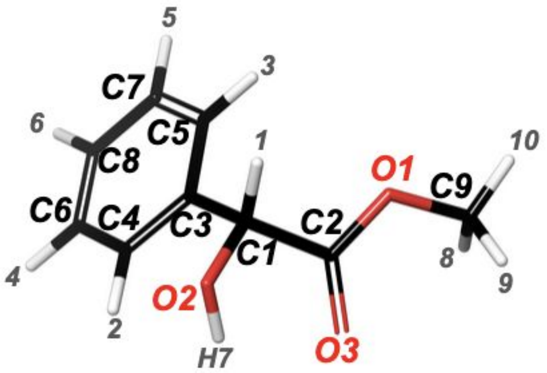

Methyl mandelate

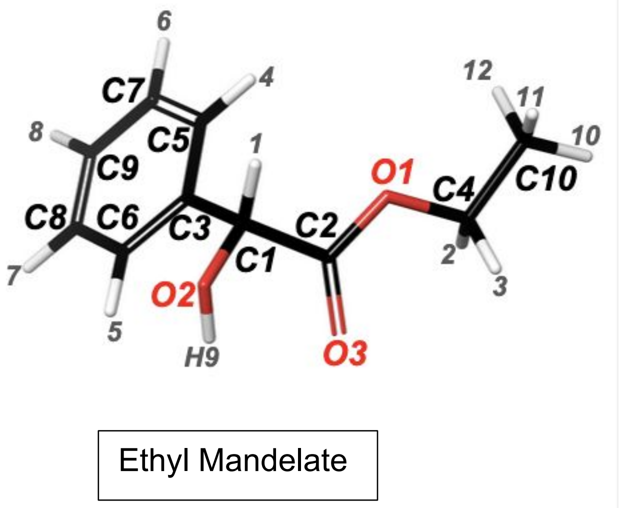

Figure S16. Atom numbering for each molecule involved in the studied DES with structures presented to scale.

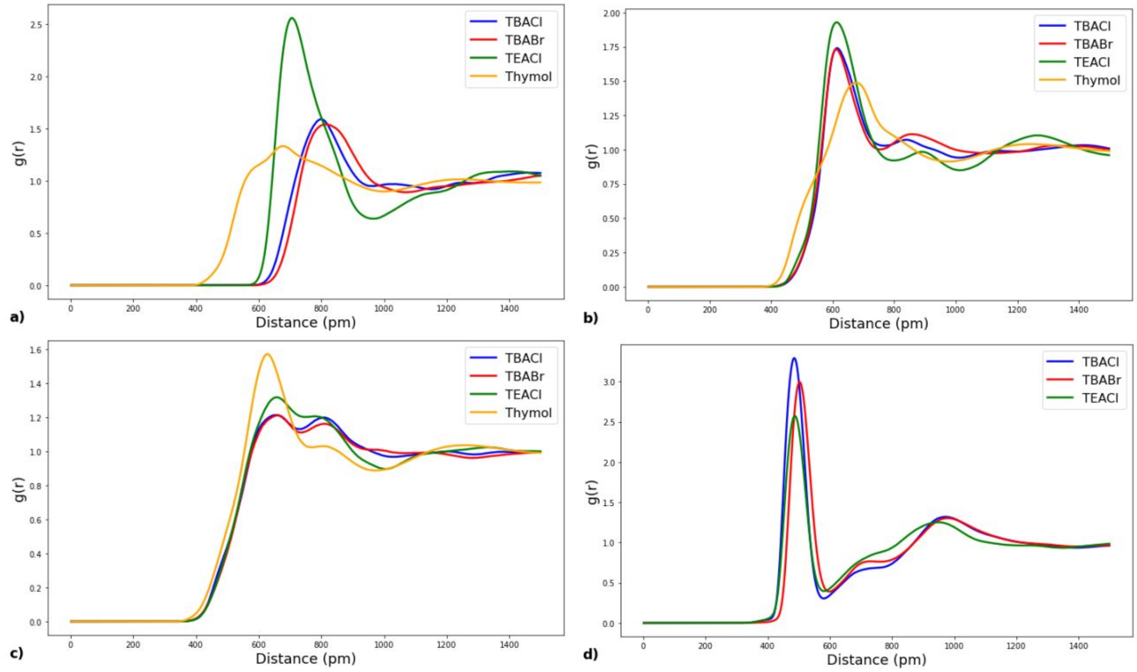

Figure S17. Center of mass radial distribution functions for the 1:2 TBABr:EM (red), TBACl:EM (blue), TEACl:EM (green) and thymol:EM (orange) DES showing a) cation-cation or thymol-thymol, b) cation-EM or thymol-EM, c) EM-EM, and d) EM-anion interactions.

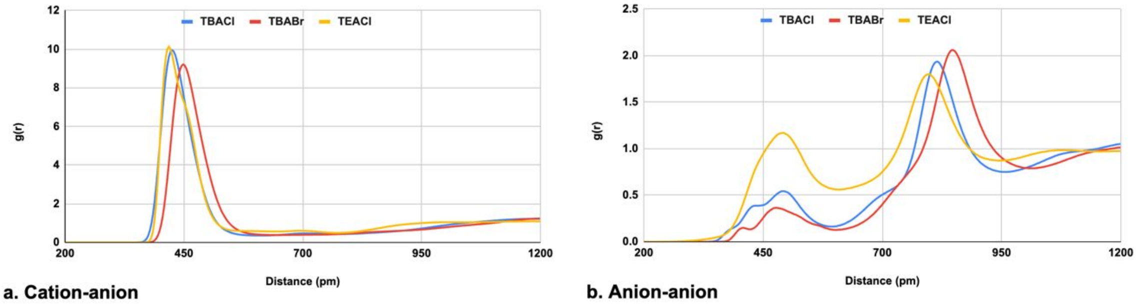

Figure S18. Center of mass radial distribution functions for the 1:2 TBABr:EM, TBACl:EM, and TEACl:EM mixtures showing a) cation-anion and b) anion-anion interactions.

Table S8. Coordination numbers for COM RDFs for HBA:EM DES.

HBA	TEACl		TBACl		TBABr	
	r_{max} (pm)	N_{coord}	r_{max} (pm)	N_{coord}	r_{max} (pm)	N_{coord}
Cation-cation	702	4.5	788	2.7	812	3.1
Cation-EM	625	5.4	612	3.0	612	3.0
Cation-anion	418	2.6	428	1.9	448	2.0
EM-EM	635	3.5	668	2.8	622	2.2
EM-anion	488	0.8	488	0.8	502	0.7
Anion-anion[a]	472	0.4	-	-	-	-
HBA	Thymol					
	r_{max} (pm)	N_{coord}				
Thymol-thymol	675	1.3				
Thymol-EM	678	1.5				
EM-EM	628	1.5				

[a] the peak maxima and minima are not well-defined in the anion-anion RDFs.

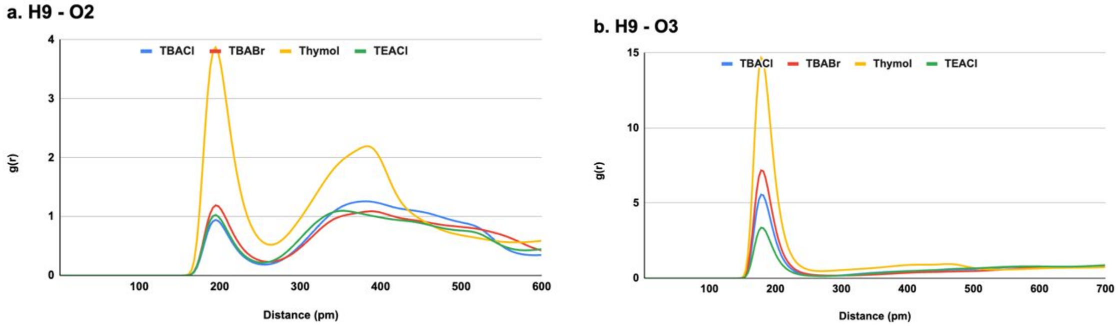

Figure S19. Atom specific RDFs that show hydrogen bonding interactions in 1:2 TBABr:EM, TBACl:EM, TEACl:EM and thymol:EM DES for a) H9– O2 (both EM) and b) H9-O3 (both EM). See Figure S16 for atom numbering.

Table S9. Results from dynamic hydrogen bond analysis of the simulations for HBA:EM DES.

	TBACl			TBABr		
	τ_f (ns)[a]	τ_b (ns)[a]	% time bonded[b]	τ_f (ns)	τ_b (ns)	% time bonded
H9-anion	13.80	0.27	98.1	3.75	0.16	95.9
Hinner-O2	5.30	3.57	59.8	4.44	3.05	59.3
Hinner-O3	5.63	2.34	70.7	7.04	3.21	68.7
	TEACl			Thymol		
	τ_f (ns)	τ_b (ns)	% time bonded	τ_f (ns)	τ_b (ns)	% time bonded
H9-anion (H9-thymol OT)[a]	13.55	0.38	97.3	0.47	1.46	24.2
Hinner-O2 (H5-O2)[a]	6.43	6.77	48.7	0.73	0.59	55.4
Hinner-O3 (H5-O3)[a]	7.88	6.11	56.4	1.05	0.54	66.3

[a] τ_f is the lifetime of hydrogen bond existence and τ_b is the lifetime of hydrogen bond formation.
[b] % time bonded = $\tau_f / (\tau_f + \tau_b)$

The NAMD stream files used to define the CHARMM format topology data structure and supply estimated missing force field parameters. These are here included for the HBD components R-ethylmandelate (**REM**), S-ethylmandelate (**SEM**) and R-methylmandelate (**RMM**); Thymol (**TMOL**); the cation component Tetra-Butyl Amine (**TBA**); and the lanthanide complex ligand 2,6-pyridine dicarboxylate anion (**DPA**). For each of these, the CGenFF website facilities (version 2.5) were used to build a CHARMM format topology file, and assign atom types, charges, and missing parameters.
The cation component Tetra-Ethyl Amine (**TEA**) already exists specifically parameterized in the CGenFF, version 4.6.

```
* Toppar stream file generated by
* CHARMM General Force Field (CGenFF) program version 2.5
* For use with CGenFF version 4.6
*

read rtf card append
* Topologies generated by
* CHARMM General Force Field (CGenFF) program version 2.5
*
36 1

! "penalty" is the highest penalty score of the associated parameters.
! Penalties lower than 10 indicate the analogy is fair; penalties between 10
! and 50 mean some basic validation is recommended; penalties higher than
! 50 indicate poor analogy and mandate extensive validation/optimization.

RESI REM            0.000 ! param penalty=   0.000 ; charge penalty=   0.377
GROUP             ! CHARGE   CH_PENALTY
ATOM O1     OG302  -0.477 !    0.239
ATOM O2     OG311  -0.638 !    0.000
ATOM O3     OG2D1  -0.621 !    0.000
ATOM C4     CG311   0.079 !    0.377
ATOM C5     CG2R61 -0.092 !    0.000
```

```
ATOM C6     CG2R61 -0.071 !    0.000
ATOM C7     CG2R61 -0.071 !    0.000
ATOM C8     CG2O2   0.894 !    0.371
ATOM C9     CG2R61 -0.115 !    0.000
ATOM C10    CG2R61 -0.115 !    0.000
ATOM C11    CG321   0.086 !    0.213
ATOM C12    CG2R61 -0.115 !    0.000
ATOM C13    CG331  -0.274 !    0.000
ATOM H14    HGA1    0.090 !    0.000
ATOM H15    HGR61   0.115 !    0.000
ATOM H16    HGR61   0.115 !    0.000
ATOM H17    HGR61   0.115 !    0.000
ATOM H18    HGR61   0.115 !    0.000
ATOM H19    HGA2    0.090 !    0.000
ATOM H20    HGA2    0.090 !    0.000
ATOM H21    HGR61   0.115 !    0.000
ATOM H22    HGP1    0.415 !    0.000
ATOM H23    HGA3    0.090 !    0.000
ATOM H24    HGA3    0.090 !    0.000
ATOM H25    HGA3    0.090 !    0.000

BOND O1   C8
BOND O1   C11
BOND O2   C4
BOND O2   H22
BOND O3   C8
BOND C4   C5
BOND C4   C8
BOND C4   H14
BOND C5   C6
BOND C5   C7
BOND C6   C9
BOND C6   H15
BOND C7   C10
BOND C7   H16
BOND C9   C12
BOND C9   H17
BOND C10  C12
BOND C10  H18
BOND C11  C13
BOND C11  H19
BOND C11  H20
BOND C12  H21
BOND C13  H23
BOND C13  H24
BOND C13  H25
IMPR C8     C4     O3     O1

END

read param card flex append
* Parameters generated by analogy by
* CHARMM General Force Field (CGenFF) program version 2.5
*

! Penalties lower than 10 indicate the analogy is fair; penalties between 10
! and 50 mean some basic validation is recommended; penalties higher than
! 50 indicate poor analogy and mandate extensive validation/optimization.

BONDS

ANGLES
```

```
DIHEDRALS

IMPROPERS

END
RETURN
```

```
* Toppar stream file generated by
* CHARMM General Force Field (CGenFF) program version 2.5
* For use with CGenFF version 4.6
*

read rtf card append
* Topologies generated by
* CHARMM General Force Field (CGenFF) program version 2.5
*
36 1

! "penalty" is the highest penalty score of the associated parameters.
! Penalties lower than 10 indicate the analogy is fair; penalties between 10
! and 50 mean some basic validation is recommended; penalties higher than
! 50 indicate poor analogy and mandate extensive validation/optimization.

RESI SEM            0.000 ! param penalty=   0.000 ; charge penalty=   0.377
GROUP            ! CHARGE   CH_PENALTY
ATOM O1     OG302  -0.477 !    0.239
ATOM O2     OG311  -0.638 !    0.000
ATOM O3     OG2D1  -0.621 !    0.000
ATOM C4     CG311   0.079 !    0.377
ATOM C5     CG2R61 -0.092 !    0.000
ATOM C6     CG2R61 -0.071 !    0.000
ATOM C7     CG2R61 -0.071 !    0.000
ATOM C8     CG2O2   0.894 !    0.371
ATOM C9     CG2R61 -0.115 !    0.000
ATOM C10    CG2R61 -0.115 !    0.000
ATOM C11    CG321   0.086 !    0.213
ATOM C12    CG2R61 -0.115 !    0.000
ATOM C13    CG331  -0.274 !    0.000
ATOM H14    HGA1    0.090 !    0.000
ATOM H15    HGR61   0.115 !    0.000
ATOM H16    HGR61   0.115 !    0.000
ATOM H17    HGR61   0.115 !    0.000
ATOM H18    HGR61   0.115 !    0.000
ATOM H19    HGA2    0.090 !    0.000
ATOM H20    HGA2    0.090 !    0.000
ATOM H21    HGR61   0.115 !    0.000
ATOM H22    HGP1    0.415 !    0.000
ATOM H23    HGA3    0.090 !    0.000
ATOM H24    HGA3    0.090 !    0.000
ATOM H25    HGA3    0.090 !    0.000

BOND O1   C8
BOND O1   C11
BOND O2   C4
BOND O2   H22
BOND O3   C8
BOND C4   C5
BOND C4   C8
BOND C4   H14
BOND C5   C6
```

```
BOND C5   C7
BOND C6   C9
BOND C6   H15
BOND C7   C10
BOND C7   H16
BOND C9   C12
BOND C9   H17
BOND C10  C12
BOND C10  H18
BOND C11  C13
BOND C11  H19
BOND C11  H20
BOND C12  H21
BOND C13  H23
BOND C13  H24
BOND C13  H25
IMPR C8     C4     O3     O1

END

read param card flex append
* Parameters generated by analogy by
* CHARMM General Force Field (CGenFF) program version 2.5
*

! Penalties lower than 10 indicate the analogy is fair; penalties between 10
! and 50 mean some basic validation is recommended; penalties higher than
! 50 indicate poor analogy and mandate extensive validation/optimization.

BONDS

ANGLES

DIHEDRALS

IMPROPERS

END
RETURN
```

```
* Toppar stream file generated by
* CHARMM General Force Field (CGenFF) program version 4.0
* For use with CGenFF version 4.6
*

read rtf card append
* Topologies generated by
* CHARMM General Force Field (CGenFF) program version 4.0
*
36 1

! "penalty" is the highest penalty score of the associated parameters.
! Penalties lower than 10 indicate the analogy is fair; penalties between 10
! and 50 mean some basic validation is recommended; penalties higher than
! 50 indicate poor analogy and mandate extensive validation/optimization.

RESI RMM           0.000 ! param penalty=   0.000 ; charge penalty=   0.000
GROUP            ! CHARGE   CH_PENALTY
ATOM O1     OG302  -0.491 !    0.000
ATOM O2     OG311  -0.638 !    0.000
```

```
ATOM O3     OG2D1  -0.630 !    0.000
ATOM C4     CG311   0.141 !    0.000
ATOM C5     CG2R61 -0.092 !    0.000
ATOM C6     CG2R61 -0.071 !    0.000
ATOM C7     CG2R61 -0.071 !    0.000
ATOM C8     CG2O2   0.859 !    0.000
ATOM C9     CG2R61 -0.115 !    0.000
ATOM C10    CG2R61 -0.115 !    0.000
ATOM C11    CG331  -0.012 !    0.000
ATOM C12    CG2R61 -0.115 !    0.000
ATOM H14    HGA1    0.090 !    0.000
ATOM H15    HGR61   0.115 !    0.000
ATOM H16    HGR61   0.115 !    0.000
ATOM H17    HGR61   0.115 !    0.000
ATOM H18    HGR61   0.115 !    0.000
ATOM H19    HGA3    0.090 !    0.000
ATOM H20    HGA3    0.090 !    0.000
ATOM H21    HGR61   0.115 !    0.000
ATOM H22    HGP1    0.415 !    0.000
ATOM HC11   HGA3    0.090 !    0.000

BOND O1   C8
BOND O1   C11
BOND O2   C4
BOND O2   H22
BOND O3   C8
BOND C4   C5
BOND C4   C8
BOND C4   H14
BOND C5   C6
BOND C5   C7
BOND C6   C9
BOND C6   H15
BOND C7   C10
BOND C7   H16
BOND C9   C12
BOND C9   H17
BOND C10  C12
BOND C10  H18
BOND C11  H19
BOND C11  H20
BOND C11  HC11
BOND C12  H21
IMPR C8     C4     O3     O1

END

read param card flex append
* Parameters generated by analogy by
* CHARMM General Force Field (CGenFF) program version 4.0
*

! Penalties lower than 10 indicate the analogy is fair; penalties between 10
! and 50 mean some basic validation is recommended; penalties higher than
! 50 indicate poor analogy and mandate extensive validation/optimization.

BONDS

ANGLES

DIHEDRALS

IMPROPERS
```

```
END
RETURN
```

```
* Toppar stream file generated by
* CHARMM General Force Field (CGenFF) program version 4.0
* For use with CGenFF version 4.6
*

read rtf card append
* Topologies generated by
* CHARMM General Force Field (CGenFF) program version 4.0
*
36 1

! "penalty" is the highest penalty score of the associated parameters.
! Penalties lower than 10 indicate the analogy is fair; penalties between 10
! and 50 mean some basic validation is recommended; penalties higher than
! 50 indicate poor analogy and mandate extensive validation/optimization.

RESI TMOL           0.000 ! param penalty=   0.000 ; charge penalty=   0.000
GROUP             ! CHARGE   CH_PENALTY
ATOM O      OG311  -0.531 !    0.000
ATOM C1     CG311  -0.090 !    0.000
ATOM C2     CG2R61 -0.006 !    0.000
ATOM C3     CG331  -0.269 !    0.000
ATOM C4     CG331  -0.269 !    0.000
ATOM C5     CG2R61  0.093 !    0.000
ATOM C6     CG2R61 -0.132 !    0.000
ATOM C7     CG2R61  0.029 !    0.000
ATOM C8     CG2R61 -0.112 !    0.000
ATOM C9     CG2R61 -0.114 !    0.000
ATOM C10    CG331  -0.264 !    0.000
ATOM H1     HGA1    0.090 !    0.000
ATOM H2     HGA3    0.090 !    0.000
ATOM H3     HGA3    0.090 !    0.000
ATOM H4     HGA3    0.090 !    0.000
ATOM H5     HGA3    0.090 !    0.000
ATOM H6     HGA3    0.090 !    0.000
ATOM H7     HGA3    0.090 !    0.000
ATOM H8     HGR61   0.115 !    0.000
ATOM H9     HGR61   0.115 !    0.000
ATOM H10    HGR61   0.115 !    0.000
ATOM H11    HGA3    0.090 !    0.000
ATOM H12    HGA3    0.090 !    0.000
ATOM H13    HGA3    0.090 !    0.000
ATOM H14    HGP1    0.420 !    0.000

BOND O    C5
BOND O    H14
BOND C1   C2
BOND C1   C3
BOND C1   C4
BOND C1   H1
BOND C2   C5
BOND C2   C6
BOND C3   H2
BOND C3   H3
BOND C3   H4
BOND C4   H5
```

```
BOND C4   H6
BOND C4   H7
BOND C5   C8
BOND C6   C9
BOND C6   H8
BOND C7   C8
BOND C7   C9
BOND C7   C10
BOND C8   H9
BOND C9   H10
BOND C10  H11
BOND C10  H12
BOND C10  H13

END

read param card flex append
* Parameters generated by analogy by
* CHARMM General Force Field (CGenFF) program version 4.0
*

! Penalties lower than 10 indicate the analogy is fair; penalties between 10
! and 50 mean some basic validation is recommended; penalties higher than
! 50 indicate poor analogy and mandate extensive validation/optimization.

BONDS

ANGLES

DIHEDRALS

IMPROPERS

END
RETURN
```

```
* Toppar stream file generated by
* CHARMM General Force Field (CGenFF) program version 2.5
* For use with CGenFF version 4.6
*

read rtf card append
* Topologies generated by
* CHARMM General Force Field (CGenFF) program version 2.5
*
36 1

! "penalty" is the highest penalty score of the associated parameters.
! Penalties lower than 10 indicate the analogy is fair; penalties between 10
! and 50 mean some basic validation is recommended; penalties higher than
! 50 indicate poor analogy and mandate extensive validation/optimization.

RESI TBA           1.000 ! param penalty=   0.900 ; charge penalty=   0.376
GROUP            ! CHARGE   CH_PENALTY
ATOM N      NG3P0  -0.592 !    0.156
ATOM C1     CG324  -0.159 !    0.219
ATOM C2     CG324  -0.159 !    0.219
ATOM C3     CG324  -0.159 !    0.219
ATOM C4     CG324  -0.159 !    0.219
ATOM C5     CG321  -0.224 !    0.376
```

```
ATOM C6      CG321  -0.224 !     0.376
ATOM C7      CG321  -0.224 !     0.376
ATOM C8      CG321  -0.224 !     0.376
ATOM C9      CG321  -0.093 !     0.343
ATOM C10     CG321  -0.093 !     0.343
ATOM C11     CG321  -0.093 !     0.343
ATOM C12     CG321  -0.093 !     0.343
ATOM C13     CG331  -0.256 !     0.304
ATOM C14     CG331  -0.256 !     0.304
ATOM C15     CG331  -0.256 !     0.304
ATOM C16     CG331  -0.256 !     0.304
ATOM H1      HGP5    0.250 !     0.000
ATOM H2      HGP5    0.250 !     0.000
ATOM H3      HGP5    0.250 !     0.000
ATOM H4      HGP5    0.250 !     0.000
ATOM H5      HGP5    0.250 !     0.000
ATOM H6      HGP5    0.250 !     0.000
ATOM H7      HGP5    0.250 !     0.000
ATOM H8      HGP5    0.250 !     0.000
ATOM H9      HGA2    0.090 !     0.000
ATOM H10     HGA2    0.090 !     0.000
ATOM H11     HGA2    0.090 !     0.000
ATOM H12     HGA2    0.090 !     0.000
ATOM H13     HGA2    0.090 !     0.000
ATOM H14     HGA2    0.090 !     0.000
ATOM H15     HGA2    0.090 !     0.000
ATOM H16     HGA2    0.090 !     0.000
ATOM H17     HGA2    0.090 !     0.000
ATOM H18     HGA2    0.090 !     0.000
ATOM H19     HGA2    0.090 !     0.000
ATOM H20     HGA2    0.090 !     0.000
ATOM H21     HGA2    0.090 !     0.000
ATOM H22     HGA2    0.090 !     0.000
ATOM H23     HGA2    0.090 !     0.000
ATOM H24     HGA2    0.090 !     0.000
ATOM H25     HGA3    0.090 !     0.000
ATOM H26     HGA3    0.090 !     0.000
ATOM H27     HGA3    0.090 !     0.000
ATOM H28     HGA3    0.090 !     0.000
ATOM H29     HGA3    0.090 !     0.000
ATOM H30     HGA3    0.090 !     0.000
ATOM H31     HGA3    0.090 !     0.000
ATOM H32     HGA3    0.090 !     0.000
ATOM H33     HGA3    0.090 !     0.000
ATOM H34     HGA3    0.090 !     0.000
ATOM H35     HGA3    0.090 !     0.000
ATOM H36     HGA3    0.090 !     0.000

BOND H34  C16
BOND H36  C16
BOND C16  H35
BOND C16  C12
BOND H24  C12
BOND H15  C8
BOND H21  C11
BOND C12  C8
BOND C12  H23
BOND H22  C11
BOND H5   C3
BOND H33  C15
BOND C8   H16
BOND C8   C4
BOND H6   C3
```

```
BOND C11  C15
BOND C11  C7
BOND C3   C7
BOND C3   N
BOND H8   C4
BOND C15  H32
BOND C15  H31
BOND C4   N
BOND C4   H7
BOND C7   H14
BOND C7   H13
BOND H11  C6
BOND H1   C1
BOND N    C1
BOND N    C2
BOND H12  C6
BOND H28  C14
BOND C6   C2
BOND C6   C10
BOND C1   H2
BOND C1   C5
BOND H29  C14
BOND C14  C10
BOND C14  H30
BOND C2   H4
BOND C2   H3
BOND H10  C5
BOND H17  C9
BOND C10  H20
BOND C10  H19
BOND C5   C9
BOND C5   H9
BOND C9   H18
BOND C9   C13
BOND H26  C13
BOND C13  H27
BOND C13  H25

END

read param card flex append
* Parameters generated by analogy by
* CHARMM General Force Field (CGenFF) program version 2.5
*

! Penalties lower than 10 indicate the analogy is fair; penalties between 10
! and 50 mean some basic validation is recommended; penalties higher than
! 50 indicate poor analogy and mandate extensive validation/optimization.

BONDS

ANGLES

DIHEDRALS
CG321  CG324  NG3P0  CG324 0.2600 3 0.00 ! TBA , from CG331 CG324 NG3P0 CG324, penalty= 0.9

IMPROPERS

END
RETURN
```

```
* Toppar stream file generated by
* CHARMM General Force Field (CGenFF) program version 2.4.0
* For use with CGenFF version 4.4
*

read rtf card append
* Topologies generated by
* CHARMM General Force Field (CGenFF) program version 2.4.0
*
36 1

! "penalty" is the highest penalty score of the associated parameters.
! Penalties lower than 10 indicate the analogy is fair; penalties between 10
! and 50 mean some basic validation is recommended; penalties higher than
! 50 indicate poor analogy and mandate extensive validation/optimization.

RESI DPA           -2.000 ! param penalty=  15.000 ; charge penalty=  29.838
GROUP             ! CHARGE   CH_PENALTY
ATOM N1     NG2R60 -0.162 !   29.838
ATOM C2     CG2R61  0.036 !   21.038
ATOM C3     CG2R61 -0.130 !    0.000
ATOM C4     CG2R61 -0.107 !    0.000
ATOM C5     CG2R61 -0.130 !    0.000
ATOM C6     CG2R61  0.036 !   21.038
ATOM H7     HGR61   0.115 !    0.000
ATOM H8     HGR61   0.115 !    0.000
ATOM H9     HGR61   0.115 !    0.000
ATOM C10    CG2O3   0.576 !    5.567
ATOM O11    OG2D2  -0.760 !    2.250
ATOM O12    OG2D2  -0.760 !    2.250
ATOM C13    CG2O3   0.576 !    5.567
ATOM O14    OG2D2  -0.760 !    2.250
ATOM O15    OG2D2  -0.760 !    2.250

BOND N1   C2
BOND N1   C6
BOND C2   C3
BOND C2   C10
BOND C3   C4
BOND C3   H7
BOND C4   C5
BOND C4   H8
BOND C5   C6
BOND C5   H9
BOND C6   C13
BOND C10  O11
BOND C10  O12
BOND C13  O14
BOND C13  O15
IMPR C10    O12    O11    C2
IMPR C13    O15    O14    C6

END

read param card flex append
* Parameters generated by analogy by
* CHARMM General Force Field (CGenFF) program version 2.4.0
*

! Penalties lower than 10 indicate the analogy is fair; penalties between 10
! and 50 mean some basic validation is recommended; penalties higher than
! 50 indicate poor analogy and mandate extensive validation/optimization.
```

```
BONDS

ANGLES
CG2O3  CG2R61 NG2R60   69.75    115.90 ! DPA, from CG2O1 CG2R61 NG2R60, penalty= 7.5

DIHEDRALS
OG2D2  CG2O3  CG2R61 NG2R60 1.9400 2 180.00 ! DPA, from OG2D1 CG2O1 CG2R61 NG2R60, penalty= 15
CG2O3  CG2R61 NG2R60 CG2R61 8.4400 2 180.00 ! DPA, from CG2O1 CG2R61 NG2R60 CG2R61, penalty= 7.5

IMPROPERS

END
RETURN
```
